# How neurons maintain their axons long-term: an integrated view of axon biology and pathology

**DOI:** 10.3389/fnins.2023.1236815

**Published:** 2023-07-26

**Authors:** Gaynor Smith, Sean T. Sweeney, Cahir J. O’Kane, Andreas Prokop

**Affiliations:** ^1^Cardiff University, School of Medicine, College of Biomedical and Life Sciences, Cardiff, United Kingdom; ^2^Department of Biology, University of York and York Biomedical Research Institute, York, United Kingdom; ^3^Department of Genetics, University of Cambridge, Cambridge, United Kingdom; ^4^Manchester Academic Health Science Centre, Faculty of Biology, Medicine and Health, School of Biology, The University of Manchester, Manchester, United Kingdom

**Keywords:** neurodegeneration, axons, axonopathies, organelles, microtubules

## Abstract

Axons are processes of neurons, up to a metre long, that form the essential biological cables wiring nervous systems. They must survive, often far away from their cell bodies and up to a century in humans. This requires self-sufficient cell biology including structural proteins, organelles, and membrane trafficking, metabolic, signalling, translational, chaperone, and degradation machinery—all maintaining the homeostasis of energy, lipids, proteins, and signalling networks including reactive oxygen species and calcium. Axon maintenance also involves specialised cytoskeleton including the cortical actin-spectrin corset, and bundles of microtubules that provide the highways for motor-driven transport of components and organelles for virtually all the above-mentioned processes. Here, we aim to provide a conceptual overview of key aspects of axon biology and physiology, and the homeostatic networks they form. This homeostasis can be derailed, causing axonopathies through processes of ageing, trauma, poisoning, inflammation or genetic mutations. To illustrate which malfunctions of organelles or cell biological processes can lead to axonopathies, we focus on axonopathy-linked subcellular defects caused by genetic mutations. Based on these descriptions and backed up by our comprehensive data mining of genes linked to neural disorders, we describe the ‘dependency cycle of local axon homeostasis’ as an integrative model to explain why very different causes can trigger very similar axonopathies, providing new ideas that can drive the quest for strategies able to battle these devastating diseases.

## The need for an integrated view of axonal cell biology

Axons are the cellular processes of neurons which form the biological cables that wire nervous systems through processes of signal propagation and synaptic transmission. In humans, axons can be as short as tens of micrometres and as long as 2 m, with diameters ranging from ~0.3 to ~15 μm depending on axon type (Prokop, 2020). These delicate structures are constantly exposed to mechanical challenges: imposed from the outside when moving our bodies, and from the inside when large cargoes are transported up and down the axon.

Nevertheless, these delicate structures must perform their highly demanding functions usually for an organism’s lifetime—up to a century in humans. Unsurprisingly, mammals lose about 40% of their axons during normal ageing (Marner et al., 2003; Adalbert and Coleman, 2012; Calkins, 2013), and many more in neurodegenerative diseases (Hopkins et al., 2021). In these conditions, the pathological decay of axons or axon segments (from now on referred to as ‘axonopathies’) is often amongst the first symptoms of neurodegeneration, not only in diseases affecting long axons in spinal cord and peripheral nerves, but also during degeneration in the brain involving much shorter axons (Stokin et al., 2005; Cheng et al., 2010).

Curiously, it appears that pathologies affecting such different axon types often display fundamental commonalities in their phenotypes, such as defects of axonal transport or a dying back from their distal synaptic terminals (Coleman, 2005; Yaron and Schuldiner, 2016; Prior et al., 2017). Even more, different types of the same class of neurodegenerative disease can be caused by mutations in genes that link to a wide range of very different cell biological processes or functions (Supplementary Table S1). Vice versa, different mutant alleles of the same gene may cause very different neural disorders (columns 4–6 in Supplementary Table S1; see for example DCTN1: Cronin and Schwarz, 2012). Such complex linkage patterns enormously complicate the search for a common disease cause. To find explanations for these rather puzzling facts, it might be helpful to explore the topic not only from the disease angle, but also use a complementary strategy by asking the fundamental question of how axons can survive and stay functional for an organism’s lifetime.

**Figure 1:**
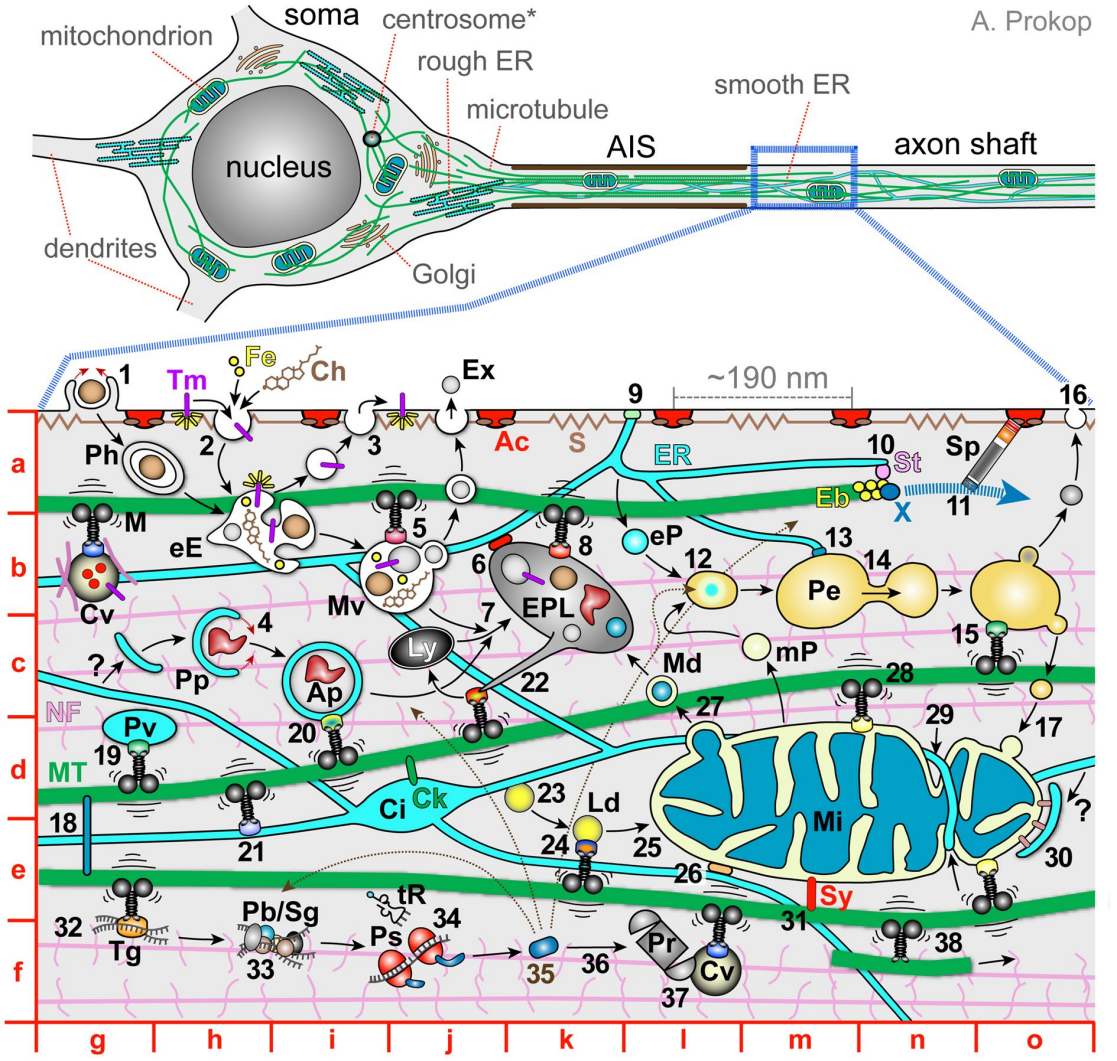
The complex network of co-existing and mutually dependent components of axonal cell biology. Top images show a schematic drawing of a typical vertebrate inter- or motorneuron (modified from Prokop, 2020) with the soma, axon initial segment (AIS) and axon shaft (further details indicated as grey text). The image below shows a magnified view of the axon shaft containing subcellular details further explained in the main text; red letters indicate map quadrants referred to in the main text. Ac, cortical actin rings; Ap, autophagosome; Ch, cholesterol; Ci, ER cisterna; Ck, CKAP4/CLIMP63 anchoring ER (Farías et al., 2019; Zheng et al., 2021); Cv, cargo vesicle carrying extracellular proteins (red), transmembrane proteins (magenta) and/or ‘hitchhiking’ cytoplasmic proteins (pink), transported by motor proteins and linker complexes; Eb, end-binding scaffold proteins; eE, early endosome; eP, ER-derived pre-peroxisome; EPL, endo-phago-lysosome; ER, endoplasmic reticulum; Ex, exosome; Fe, iron; Ly, lysosome; M, motor protein (throughout the image motors are in black, their cargo adaptors shown as differently coloured dots, and vibration lines represent mechanical challenges to MTs); Md, mitochondria-derived vesicle; Mi, mitochondrion; MT, microtubules; mP, mitochondria-derived pre-peroxisome; Mv, late endosome or multivesicular bodies; NF, neurofilaments with sidearms; Pb, P-body; Pe, peroxisome; Ph, phagosome; Pp, phagophore; Pr, proteasome; Ps, polysome; S, cortical spectrin with spring-like properties; ER; Sg, stress granule; Sp, spectraplakins guiding the extension of polymerising MTs; St, STIM1; Sy, syntaphilin; Tg, transport ribonucleoprotein granule; Tm, transmembrane prote (yellow rays indicate signalling activity); tR, transfer RNA; X, XMAP215 polymerase; ?, potential origin of phagophores from the ER. Explanations of numbers: **1**, phagocytosis; **2**, endocytosis of surface proteins; **3**, exocytosis to release extracellular proteins or re-insert surface proteins (a combination of ‘2’ and ‘3’ makes up transcytosis as an indirect transport mechanism; Cornejo et al., 2017); **4**, incorporation of protein aggregates by phagophores; **5**, transport of endosomes; **6**, lysosomal-ER membrane contact site; **7**, late endosomes and autophagosomes fuse with lysosomes for degradation; **8**, transport of lysosomes; **9**, ER-plasma membrane contact site (details in Figure 3C); **10**, STIM1-mediated linkage of ER tips to polymerising MT plus ends; **11**, extension of polymerising MT plus ends guided by Sp; **12**, mitochondria- and ER-derived pre-peroxisomes fuse into mature peroxisomes; **13**, ER/peroxisome contacts to maintain peroxisomes and lipid homeostasis; **14**, budding of peroxisomes; **15**, peroxisome transport; **16**, peroxisome provision of lipids to the plasma membrane; **17**, delivery of peroxisome-derived mitochondrial lipids; **18**, cross-linkage of MTs via MT lattice-binding proteins (e.g., MAPT = tau, MAP1B) or mitotic kinesins; **19**, vesicular transport of lipids (plasmalemmal precursor vesicles; PPVs); **20**, autophagosome transport; **21**, transport of ER; **22**, tubulation of endo-phago-lysosomes to restore lysosomes (Du et al., 2016); **23**, formation of lipid droplets; **24**, lipid droplet transport (Kilwein and Welte, 2019); **25**, lipid provision to organelles; **26**, ER-mitochondrial membrane contact site (details in Figure 3C); **27**, MDV-mediated disposal of faulty proteins; **28**, axonal transport of mitochondria; **29**, fission and fusion of mitochondria supported by ER-mediated constriction; **30**, mitophagy, dependent on recruitment via PINK (PTEN-induced putative kinase) and the E3 ubiquitin ligase parkin (pink colour; Ashrafi and Schwarz, 2015); **31**, syntaphilin-mediated anchorage of mitochondria (Cardanho-Ramos et al., 2019); **32**, transport of non-vesicular cargoes such as RNA or proteins; **33**, posttranscriptional storage/regulation of messenger RNAs; **34**, local translation; **35**, newly available protein distributing to its target locations (indicated by thin black stippled arrows); **36**, degradation of cytoplasmic proteins; **37**, fast transport of proteasomes; **38**, kinesin-1 sliding/transporting of MTs along other MTs (Lu et al., 2015; Winding et al., 2016).

Answering this question has to take into account that, especially in long axons, nuclear derived factors take days and even months to be delivered to the most distal parts (axonal transport rates range from ~1 to ~240 mm/day; Grafstein and Forman, 1980). The maintenance of most if not all axons requires therefore the on-site presence of essential organelles and endomembrane specialisations including mitochondria (‘Mi’ in quadrant e/m of Figure 1), endoplasmic reticulum (‘ER’ in Figure 1 >a/l), peroxisomes (‘Pe’ in Figure 1 >b/m), lipid droplets (‘Ld’ in Figure 1 >e/k), endosomal, lysosomal and (auto-) phagosomal specialisations (‘eE, Mv, Ly, Ap’ in Figure 1 >b +c/i + j). Furthermore, axons contain cytoplasmic or plasma membrane-associated machineries that drive metabolic and signalling processes, maintain proteostasis and uphold the homeostasis of reactive oxygen species. An axon is therefore almost like a cell within a cell, which can explain how neurons of tiny parasitic wasps can maintain their axons after shedding their nuclei in the adult CNS (Polilov, 2012); or how detached distal segments of severed axons can persist for hours and days before they eventually undergo Wallerian degeneration (Rawson et al., 2014; Llobet Rosell and Neukomm, 2019).

Apart from cell-autonomous processes, the function and longevity of axons is usually also reliant on glial cells (Verkhratsky and Butt, 2007; Stassart et al., 2018): glia may ensheath them (e.g., myelination, Remak bundles), provide a signalling environment and deliver materials via exosomes, extracellular vesicles, or the lactate shuttle (Frühbeis et al., 2013; Stassart et al., 2018; Chamberlain et al., 2021; Deck et al., 2021; Mietto et al., 2021; Bastian et al., 2022; Beirowski, 2022). Accordingly, conditions of dysfunctional or absent glia can deprive axons of energy, molecular components or structural support, and disturb their electrical/synaptic properties and signalling networks; such externally caused disturbances of axonal homeostasis can eventually trigger cell-autonomous processes of axon decay (Pan and Chan, 2017; Philips et al., 2021; Licht-Mayer et al., 2022; Chuang et al., 2023).

Here, we will restrict discussions to axons and summarise key features of their most prominent subcellular components and mechanisms. We will highlight in text and illustrations the complex and interdependent networks formed by these components and mechanisms required to sustain long-lasting axonal homeostasis. In our view, the appreciation of this complexity is an important prerequisite to gain a more holistic and integrated understanding of axons which will eventually facilitate the development of strategies that can tackle the systemic medical problem of axon degeneration.

## Roles and regulations of mitochondria in axons

**Figure 2:**
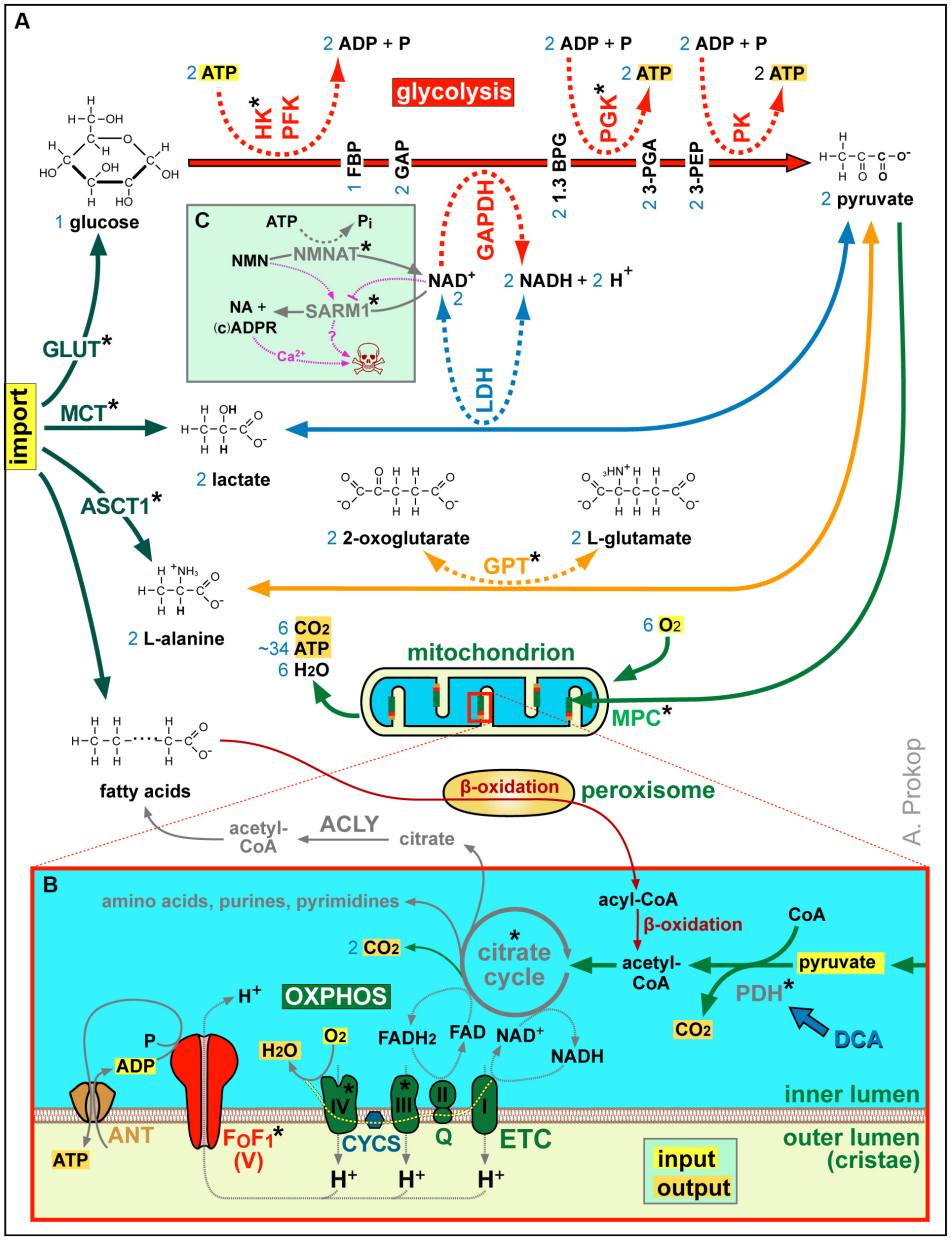
Summary of ATP production and other aspects of metabolism. **(A)** Acetyl-CoA is either a product of β-oxidation (in peroxisomes and mitochondria) or of pyruvate generated from imported L-alanine, lactate or glucose (the latter involving glycolysis as an ATP-generating mechanism that requires NAD^+^ as an oxidising agent; Gray et al., 2014). **(B)** Acetyl-CoA feeds the citrate cycle that generates FADH_2_ and NADH supplying electrons for oxidative phosphorylation (OXPHOS): the electron transfer chain (ETC) transfers protons into the outer mitochondrial lumen of cristae which drive chemiosmotic ATP synthase activity via F_0_F_1_. Metabolic side products of the citrate cycle are indicated in grey. Blue numbers in **(A,B)** refer to numbers of molecules produced or required per one molecule of glycose invested. **(C)** As revealed by studies of Wallerian degeneration in mammals and fly (Llobet Rosell and Neukomm, 2019; Figley et al., 2021; Hopkins et al., 2021; Llobet Rosell et al., 2022), loss of NMNAT increases the NMN to NAD^+^ ratio which triggers the activation of the death factor SARM1 (to which both molecules can bind competitively); the SARM1 products ADPR and cADRP trigger calcium channel openings of TRPM2 and RYR, respectively (see Figure 3C) causing axon death (skull) jointly with other events downstream of SARM1 (‘?’); blocking SARM1 activity has therefore neuroprotective potential, with first compounds emerging (e.g., Zn^2+^ or Berberine chloride; Liu et al., 2014; Negahdar et al., 2015; Trakhtenberg et al., 2018; Loring et al., 2020; Loring and Thompson, 2020). Asterisks indicate proteins with OMIM-listed links to neural disorders; all abbreviations of gene names and their existing OMIM links are listed in Supplementary Table S3.

Neurons have enormous demands for ATP: a single cortical neuron in the human brain at resting state was estimated to use ~4.7 billion ATP molecules per second (Zhu et al., 2012). The energy cost increases upon neuronal activity (Attwell and Laughlin, 2001) and with axonal arborisation and size, making some neurons more vulnerable to ATP depletion than others (Pissadaki and Bolam, 2013). ATP is needed to sustain electrical properties, and it fuels many other life-sustaining processes, such as protein assembly and degradation, actin polymerisation, MT severing, motor protein dynamics, processes of phosphorylation, and GTP production (Berg et al., 2002; Meyer et al., 2003; Bogoyevitch and Fairlie, 2007; Hall and Lalli, 2010; Zala et al., 2013; Voelzmann et al., 2016; McNally and Roll-Mecak, 2018; Skruber et al., 2018). To saturate this demand, cytoplasmic glycolysis appears insufficient (2 ATP per glucose; Figure 2A). There is a likely need for effective mitochondrial oxidative phosphorylation (OXPHOS; >30 ATP per glucose molecule; Figure 2B), and the presence of mitochondria is a prominent feature of axons (Misgeld and Schwarz, 2017). To meet ‘the energy demands of axons,’ adequate distribution of mitochondria through transport and fission/fusion dynamics is pivotal (discussed below).

As shown in Figure 2B, mitochondria perform β-oxidation or import pyruvate to generate acetyl-CoA required to drive their citrate cycle for the production, directly or indirectly, of certain amino acids, purines and pyrimidines, lipids, NADH and FADH_2_. Of these, NADH and FADH_2_ are also produced by β-oxidation, and they can also be imported via the NAD-redox shuttle (not shown in Figure 2B; Wanders et al., 2016). NADH and FADH_2_ act as electron donors that fuel the highly efficient process of OXPHOS-mediated ATP production involving two steps (Figure 2B): the electron transfer chain generates a proton gradient across the membranes of cristae which then drives the F_0_F_1_ ATP synthase as a highly efficient generator of ATP.

**Figure 3:**
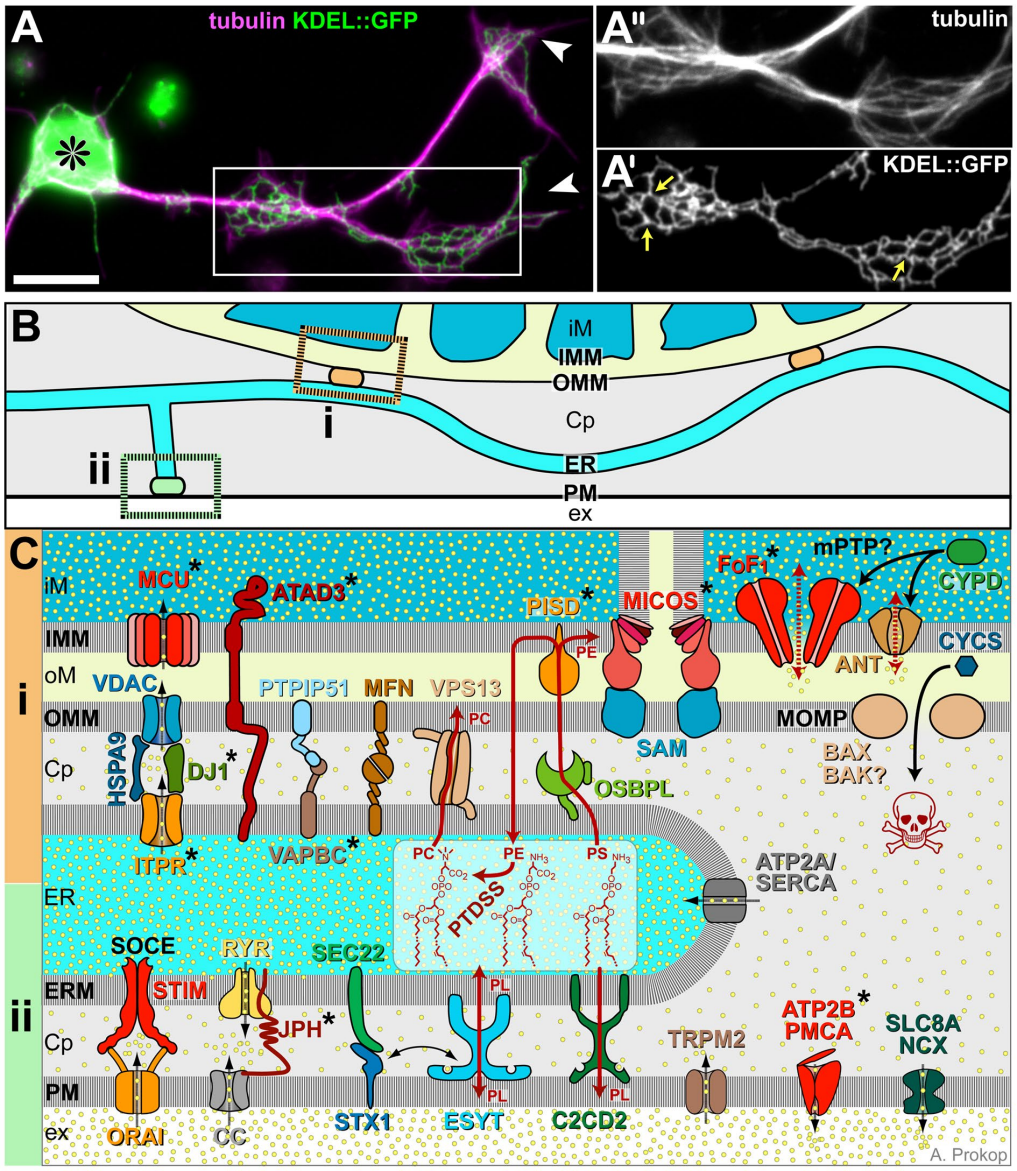
ER morphology and details of its membrane contact sites. **(A)** *Drosophila* primary neuron (asterisk, cell body; arrowhead, axon tips) stained for tubulin/MTs (magenta) and neuronally expressed KDEL::GFP to label axonal ER (Irvine, 2007; 1.24); boxed area highlights regions of axonal swelling, shown 1.4-fold enlarged as single channel images in **A′** and **A″**; yellow arrows in **A″** point at three-way junctions; scale bar in **(A)** represents 10 μm. **(B)** Illustration of ER tubules (light blue) making contacts with mitochondria (‘**i**’, orange) or plasma membrane (‘**ii**’, green; colour-code as in Figure 1). **(C)** Molecular details of ER contact sites with mitochondria at the top (‘**i**’, orange), plasma membrane at the bottom (‘**ii**’, green) and ER in between: Cp, cytoplasm; ER, ER lumen; ERM, ER membrane; ex, extracellular space; iM/oM; inner/outer mitochondrial lumen; and IMM/OMM, inner/outer mitochondrial membrane. Symbols: yellow dots, Ca^2+^ ions; black stippled arrows, flow of Ca^2+^ ions; dark red arrows, non-vesicular transfer of lipids (PC, PE, PL and PS); stippled red arrows: opening of the mPTP; solid black arrows: protein translocation: release of CYCS through the MOMP is a key event of apoptosis (skull). Asterisks indicate proteins with OMIM-listed links to neural disorders; all abbreviations of gene names and their existing OMIM links are listed in Supplementary Table S3.

Mitochondrial metabolism and ATP production are regulated by Ca^2+^ (Verstreken et al., 2005; Rossi et al., 2019), which enters mitochondria through voltage-dependent anion channels and the mitochondrial calcium uniporter complex (‘VDAC, MCU’ in Figure 3C; Baughman et al., 2011; De Stefani et al., 2011; Choi et al., 2017; Tufi et al., 2019). This import can take place at membrane contact sites with the ER, which can generate microdomains of high enough Ca^2+^ concentration to feed the MCU (Figure 3C; Raffaello et al., 2016). If mitochondrial Ca^2+^ concentrations are high, transient openings of the low-conductance mPTP (likely formed by the adenine nucleotide translocator; ‘ANT’ in Figure 3C), provide an ‘overflow valve’ permeating the inner mitochondrial membrane for small molecules including ions (Bauer and Murphy, 2020; Bonora et al., 2022).

Mitochondrion-ER membrane contact sites are systemically important: for example, enhanced axon growth was observed when artificially tethering mitochondria to the ER (Lee et al., 2019). This may be due to numerous functions that these contacts have beyond Ca^2+^ exchange: they mediate lipid exchange (details in Figure 3C), appear to be sites of mitochondrial biogenesis regulation and contribute to the regulation of mitochondrial transport and fission/fusion dynamics (Csordás et al., 2018; Laar et al., 2018; Abrisch et al., 2020; Giacomello et al., 2020; Damenti et al., 2021; Hewitt et al., 2022).

For example, to induce mitochondrial fission, ER wraps around mitochondria, believed to mediate pre-constrictions (‘29’ in Figure 1 >d/n; Friedman et al., 2011; Kraus and Ryan, 2017) that eventually lead to mitochondrial scission; the actual constriction leading to scission of the outer mitochondrial membrane involves polymerising actin networks and helical oligomers of dynamin-related protein (DRP1; Kraus et al., 2021). The cardiolipin-rich inner mitochondrial membrane requires separate fission machinery involving the dynamin GTPase family member OPA1. Alternative mechanisms of mitochondrial fission are reported to involve kinesin-driven tubulation of mitochondria (Wang et al., 2015). Apart from its involvement in fission, OPA1 is also required for the formation of cristae harbouring the electron transfer chain and F_0_F_1_ ATP synthase (Figures 2A,B), and for the fusion of the inner mitochondrial membrane; fusion of the outer membrane is mediated by further dynamin-like GTPases MFN1 and 2 (Gao and Hu, 2021). Fission and fusion regulate numbers and sizes of mitochondria essential for neuronal integrity and function (Ishihara et al., 2009; Rambold et al., 2011; Berthet et al., 2014; Kiryu-Seo et al., 2016). For example, mitochondria need to be small enough to undergo trafficking or autophagy (‘28, 30’ in Figure 1 >c/n and e/o), and their size influences mitochondrial and cytoplasmic calcium levels (Kowaltowski et al., 2019).

According to the endosymbiotic theory, mitochondria are of bacterial evolutionary origin. In consequence, mitochondrial machinery is mostly encoded by nuclear genes, but in small part also by mitochondrial genes, which do not display Mendelian genetics (Ojala et al., 1981; Wallace, 2018; Rath et al., 2020; Ribas De Pouplana, 2020; Dowling and Wolff, 2023). This means that fission and fusion processes need to be coordinated with processes of mitochondrial DNA replication (performed by POLG2) and segregation (Kraus et al., 2021). It also means that mitochondria cannot be generated anew by cells, but existing pools must be passed on during cell division, actively maintained and replicated when needed. Their replication requires local biogenesis: mitochondrial mtDNA replication occurs at the ER interface, mitochondrially encoded proteins are produced by mitoribosomes (Greber and Ban, 2016), nuclear encoded proteins are imported. The redistribution of newly grown mitochondrial networks can then occur via fission as the final step of biogenesis. However, it should be noted that fission and fusion can also act independently of biogenesis in axons, for example in stress conditions. Furthermore, mitochondrial maintenance involves their removal through autophagy (either locally or involving retrograde transport to the cell body; see endomembrane section), mitochondrial chaperone systems and AAA+ proteases, and the shedding of faulty proteins via mitochondria-derived vesicles (‘27’ in Figure 1 >d/l; Amiri and Hollenbeck, 2008; Glynn, 2017; Harbauer, 2017; Misgeld and Schwarz, 2017; Laar et al., 2018; Cardanho-Ramos and Morais, 2021; Mandal et al., 2021; Adriaenssens et al., 2023).

As well as contact sites with the ER, mitochondria also form contacts with endosomes, lysosomes and peroxisomes (not shown in Figure 1). For example, contacts with endosomes can deliver cargoes, such as cholesterol or iron to mitochondria, lysosomes and mitochondria mutually regulate their maintenance through these sites, and peroxisomes pass on short-chain acyl-CoA derived from peroxisomal β-oxidation (Figures 2A,B; Schrader et al., 2015; Wanders et al., 2016; Todkar et al., 2019; Okumoto et al., 2020; Uzor et al., 2020; Wanders et al., 2020). The close communication between mitochondria and peroxisomes seems also important to uphold the homeostasis of reactive oxygen species (ROS) and coordinate parallel processes of their fission or autophagy (Fransen et al., 2017; Pascual-Ahuir et al., 2017; Kamerkar et al., 2018).

## Roles and regulations of the ER in axons

The ER has various fundamental functions: rough ER (usually positioned around the nucleus) executes the translation of transmembrane and extracellular proteins and the initial steps of N-linked protein glycosylation, whereas the peripheral smooth ER is equipped to synthesise neutral lipids, sterols, sphingolipids and phospholipids; further important ER functions are the regulation of cytoplasmic and mitochondrial Ca^2+^ as well as gluconeogenesis (Öztürk et al., 2020).

The ER networks in axons have characteristics typical of smooth ER: they do not display extensive machinery for transmembrane or extracellular protein production, but harbour enzymes for lipid biosynthesis and metabolism, as well as channels and transporters to regulate Ca^2+^ levels (Figure 3; O'Sullivan et al., 2012; Yalçin et al., 2017; Luarte et al., 2018). Axonal ER tubules are narrow, often as thin as 10 nm, and their networks are believed to run uninterrupted along the entire length of axons (Tsukita and Ishikawa, 1976; Burton and Laveri, 1985; Terasaki et al., 1994; O'Sullivan et al., 2012; Wu et al., 2017; Yalçin et al., 2017; Terasaki, 2018; Foster et al., 2021; Hoffmann et al., 2021). In growing axons, the ER seems to take on a ladder-like appearance interspersed by occasional small sheets and irregularly shaped cisternae, but we have little current knowledge about the potential function of these specialisations (González and Couve, 2014; Zamponi et al., 2022; Zhu et al., 2022). In areas of axonal swellings, ER networks display the typical 3-way junctions giving rise to Voronoi diagram- or giraffe-like patterns, as similarly found in non-neuronal cells (Liew et al., 2021; Obara et al., 2023; Figure 3A).

To ensure continuity and uniformity of ER networks, they are dynamically maintained (for details see Öztürk et al., 2020): (1) they are dragged along with the tips of polymerising MTs (via EB-STIM1 interaction; ‘10’ in Figure 1 >a/n) or moved along MT lattices by motor proteins regulating their shape and distribution (‘21’ in Figure 1 >e/h; Farías et al., 2019; Petrova et al., 2020; Zamponi et al., 2022), (2) they can be anchored to MTs via linkers (‘Ck’ in Figure 1 >d/j; Farías et al., 2019; Zheng et al., 2021), (3) they can undergo DNM1L-mediated fission and atlastin-mediated fusion (Öztürk et al., 2020), (4) their tubular and cisternal structure is actively shaped by hairpin-loop-domain containing proteins (REEPs, reticulon, ARL6IP1, protrudin, and potentially spastin; Renvoisé and Blackstone, 2010; Yperman and Kuijpers, 2023), and (5) their three-way junctions are stabilised by lunapark (Obara et al., 2023).

Endoplasmic reticulum-mediated lipidogenesis is of particular importance for axons: ‘*an axon 0.5 μm in diameter that is growing at the rate of 0.5–1 mm per day will require the addition of 0.5–1 μm^2^ of plasma membrane per minute*’ (Landis, 1983). Accordingly, inhibition of cholesterol synthesis in cultured neurons was shown to reduce axon extension by 50% (de Chaves et al., 1997). Whether *de novo* lipidogenesis takes place in neuronal cell bodies followed by transport into axons via plasmalemmal precursor vesicles (‘Pv’ in Figure 1>d/g), or whether the axonal ER can execute this function locally remains an open debate (Pfenninger, 2009). For certain, the ER can remodel lipids and exchange them at different membrane contact sites: (a) contacts with the plasma membrane can deliver membrane lipids (Figure 3Cii and ‘9’ in Figure 1 >a/k; Gallo et al., 2020); (b) contacts with peroxisomes can pass on excess lipids for β-oxidation (‘13’ in Figure 1 >b/m; Wanders et al., 2020); (c) contacts with mitochondria can exchange lipids and process them with complementary sets of enzymes (‘26’ in Figure 1 e/l, Figure 3C; Wanders et al., 2016); (d) contacts with lipid droplets (‘Ld’ in Figure 1 >e/k) can transfer lipids for their storage (Hong et al., 2022) or as a means of cellular detoxification and protection against oxidation. This said, lipid droplets are sometimes present in axons, but they are far more prominent in glia (Pennetta and Welte, 2018; Olzmann and Carvalho, 2019; Farmer et al., 2020; Öztürk et al., 2020; Islimye et al., 2022).

The ER is the major intracellular Ca^2+^ store. It protects from calcium cytotoxicity whilst being able to release Ca^2+^ as a second messenger (Raffaello et al., 2016). For example, cytoplasmic Ca^2+^ levels can be rapidly reduced via ATP2A (= SERCA) pumps in the ER, in parallel to ATP2B/PMCA and SLC8A/NCX in the plasma membrane (all bottom right in Figure 3C), thus terminating Ca^2+^ peaks during signalling events and preventing cytotoxicity (Bagur and Hajnóczky, 2017). Stimulated Ca^2+^ release from the ER is mediated by inositol 1,4,5-triphosphate and ryanodine receptors (‘ITPR, RYR’ in Figure 3; Raffaello et al., 2016). ER-mediated Ca^2+^ regulation also involves membrane contact sites with other cellular structures. For example, upon depletion of its Ca^2+^ stores the ER can replenish them at contact sites with the plasma membrane, whereas it exports Ca^2+^ to mitochondria at mitochondrial contact sites (Figure 3C; Karagas and Venkatachalam, 2019; Öztürk et al., 2020; Pérez-Moreno et al., 2023).

Due to their uninterrupted nature in axons, ER networks are the largest axonal organelle, and have been described as a ‘neuron within a neuron’ (Berridge, 1998; Berridge et al., 2003). They seem to provide a means of regional or long-distance communication, potentially even independent of neuronal conduction at the plasma membrane. For example, during injury-induced axon regeneration of dorsal root ganglion neurons, ER-dependent long-range Ca^2+^ waves that back-propagate from the injury site to the nucleus at ~10 μm/s were required to trigger transcriptional responses (Cho et al., 2013). Ca^2+^ events mediated by ryanodine receptor stimulation were recently reported to involve ER-mediated calcium-induced calcium release and Ca^2+^ wave propagation (Campbell et al., 2023). The properties of these waves are influenced by neurite and ER diameters and RYR density (Breit and Queisser, 2018). Since the ER closely interacts with virtually all other organelles (Wenzel et al., 2022), its intra-axonal signalling function might be a means of maintaining axonal homeostasis by mediating inter-organelle communication.

## Roles and regulations of the endomembrane system in axons

In non-neuronal cells as in axons, the endo- and auto−/phagosomal systems (from now on jointly referred to as endomembrane system) provide key machinery to incorporate material in vesicles and transfer them to other cellular locations or pass them on for degradation. Endocytosis has key roles in the internalisation of transmembrane proteins (e.g., to regulate axonal growth; Hines et al., 2010; Tojima and Kamiguchi, 2015) and for the import of extracellular materials including proteins, cholesterol or iron (ferritin receptor-mediated endocytosis; ‘2, Fe, Ch’ in Figure 1 >a/h + i; e.g., Mauch et al., 2001; Mills et al., 2010; Zhang and Liu, 2015; Cornejo et al., 2017). Axons can receive most of these materials from glia (Mietto et al., 2021). Endocytosed material is shuttled towards the phosphatidylinositol 3-phosphate-enriched, RAB5-positive early endosome (‘eE’ in Figure 1 >b/h) which provides a signalling hub (yellow rays in Figure 1 >a/h; Gillooly et al., 2000; Villaseñor et al., 2016) and a site where cargo is sorted and channelled (via fusion, fission and budding processes) to distinct endosomal compartments. Each compartment is distinguished by specific lipids, acidities and/or molecular machineries (Rink et al., 2005; Poteryaev et al., 2010; Liu et al., 2016; Naslavsky and Caplan, 2018). The best established pathways of cargo shuttling are: (i) back to the surface in RAB4-positive vesicles (fast recycling; ‘3’ in Figure 1 >a/i; Dey et al., 2017); (ii) to the RAB11-positive recycling endosome for slow recycling (Rozés-Salvador et al., 2020); (iii) to the RAB7-positive late endosome (Vitelli et al., 1997) and ESCRT complex-bearing multivesicular bodies (‘Mv’ in Figure 1 >b/i; Katzmann et al., 2001; Henne et al., 2011; Schuh and Audhya, 2014; Christ et al., 2017). From the latter, cargo can be: (a) passed on to other organelles at membrane contact sites (Todkar et al., 2019); (b) shuttled to the plasma membrane and released via exosomes (‘Ex’ in Figure 1 >a/j; Jin et al., 2018); (c) channelled for degradation through fusion with LAMP-positive lysosomes (‘7’ in Figure 1 >c/j; details below). As the provided references suggest, all or most of these processes take place in axons, in addition to cell bodies.

Larger extracellular particles, such as apoptotic cell debris, are incorporated via phagocytosis through engulfment by actin-driven cup-shaped membrane protrusions (‘1’ in Figure 1 >a/g). Phagocytosis is mainly observed in glia but can occur in axons (Bowen et al., 2007; Uribe-Querol and Rosales, 2020). In contrast, auto-phagocytosis (autophagy) is the process of incorporating large objects from the cytoplasm, such as protein aggregates (aggrephagy) or entire organelles (mitophagy, pexophagy, lipophagy, lysophagy and ER-phagy; reviewed in Bento et al., 2016; Maday, 2016; Mochida and Nakatogawa, 2022; Wrobel et al., 2022). This is achieved by nascent phagophores (‘Pp’ in Figure 1 >c/h) that arise from intramembranous structures (potentially the ER; ‘?’ in Figure 1 >c/g; Bento et al., 2016) and engulf objects carrying specific ‘tags’ such as ubiquitin, ubiquilin or SQSTM1 (Bjørkøy et al., 2005; Pankiv et al., 2007). Through this process of ‘self-digestion’ materials can be recycled to support cells during episodes of starvation, and it is a means of protection by removing pathological inclusions, aggregates or damaged organelles. Unlike endosomes, ATG-positive auto−/phagosomes are enclosed by double-membranes (‘Ap, Ph’ in Figure 1 >c/i and a/g) and primarily destined for degradation.

Degradation through both the endosomal and auto−/phagosomal paths contributes to axonal proteostasis protecting from starvation and pathological threats—all essential for axonal longevity. To achieve degradation, phagosomes, but also endosomes and other membranous components (e.g., mitochondria-derived vesicles; ‘27, Md’ in Figure 1 >d/l), need to fuse with lysosomes into hybrid compartments, such as endo-lysosomes or phago-lysosomes (‘7, Ly, EPL’ in Figure 1 >b + c/j + k; Tjelle et al., 1996; Bright et al., 2016). This may occur through intermediate steps (e.g., endo-autophagosomes = amphisomes).

To facilitate degradation, lysosomes are rich in acid hydrolases (about 60 different enzymes reported, such as cathepsins, glucocerebrosidase and galactosylceramidase; Bonam et al., 2019; Berge-Seidl and Toft, 2020; Kreher et al., 2022; Roney et al., 2022) which seem to become active upon lowering of the pH when lysosomes fuse with late endosomes to form endolysosomes (Bright et al., 2016). The position of lysosomes in cells appears critical to their function: in non-neuronal cells, peripheral lysosomes are less acidic; this correlates with reduced levels of Rab7 and RILP required for the recruitment of the lysosomal proton pump, the V-ATPase complex (Johnson et al., 2016). The same might be true in distal axons.

Apart from degradation, lysosomes are also critical for sensing and responding to nutritional deprivation (Korolchuk et al., 2011; Pu et al., 2017), although it remains to be seen whether this occurs in axons or is restricted to neuronal cell bodies. Furthermore, lysosomes can fuse with the plasma membrane to initiate injury repair and/or to release enzymes for extracellular matrix modification (Andrews et al., 2014; Tancini et al., 2020). They might also contribute to Ca^2+^ signalling events, which can involve membrane contact sites with the ER (Raffaello et al., 2016). Local calcium release via endolysosome-based mucolipidins and two-pore segment channels (‘MUCOLN1, TPCN2’ in Supplementary Table S1) is known to regulate endolysosomal fusion dynamics.

Lysosomes can be recycled or reformed from endo−/auto−/phago-lysosomes involving clathrin- and kinesin-mediated tubulation of their surfaces (‘22’ in Figure 1 >c/k; Du et al., 2016; Yang and Wang, 2021), and they undergo dynamic fission/fusion events (Saffi and Botelho, 2019). *De novo* formation of lysosomes occurs through gradual fusion with hydrolase-containing, Golgi-derived late endosomal vesicles (Yang and Wang, 2021). In long axons, this requires substantial adaptations since the Golgi is restricted to the distant soma. Accordingly, mature lysosomes are primarily found in the somato-dendritic region (Parton et al., 1992) whilst degradative lysosomes in axons appear less mature or more specialised (Lee et al., 2011; Gowrishankar et al., 2015; Jin et al., 2018). The endomembrane system is therefore heavily dependent on long distance MT-based and short-range actin-based transport (‘5, 8, 20’ in Figure 1 >b/j, b/k and d/i), with individual entities often maturing during their travel (Tsukita and Ishikawa, 1980; Hollenbeck, 1993; Overly and Hollenbeck, 1996; Deinhardt et al., 2006; Schink et al., 2016; Farías et al., 2017; Farfel-Becker et al., 2019; Chakrabarti et al., 2021; Keren-Kaplan and Bonifacino, 2021; Roney et al., 2022).

Autophagosomes can form in axons, for example in response to activity at the synapse (Yang et al., 2022b), and are retrogradely transported via dynein and varying adaptor complexes (Maday et al., 2012); during their voyage, autophagosomes become increasingly acidified and enriched with cathepsins through SNARE-mediated fusion events (Kimura et al., 2007; Itakura et al., 2012; Takáts et al., 2013), until they eventually fuse with somatic lysosomes in a WDR91-dependent manner (Liu et al., 2017; Xing et al., 2021).

## Roles and regulations of peroxisomes in axons

Peroxisomes are present in axons (Wang et al., 2018) but far more prominent and relevant in glial cells (Kassmann et al., 2007; Bottelbergs et al., 2010). They can be formed *de novo* through the fusion of ER- and mitochondria-derived pre-peroxisomes, concurrent with the import of matrix proteins that contain peroxisomal targeting signals (‘12’ in Figure 1 >b/l; Kim, 2017; Sugiura et al., 2017; Fujiki et al., 2020). Once formed, peroxisomes can amplify through DNM1L (= DRP1)-mediated fission and budding (‘14’ in Figure 1 >b/n; Fujiki et al., 2020).

**Figure 4:**
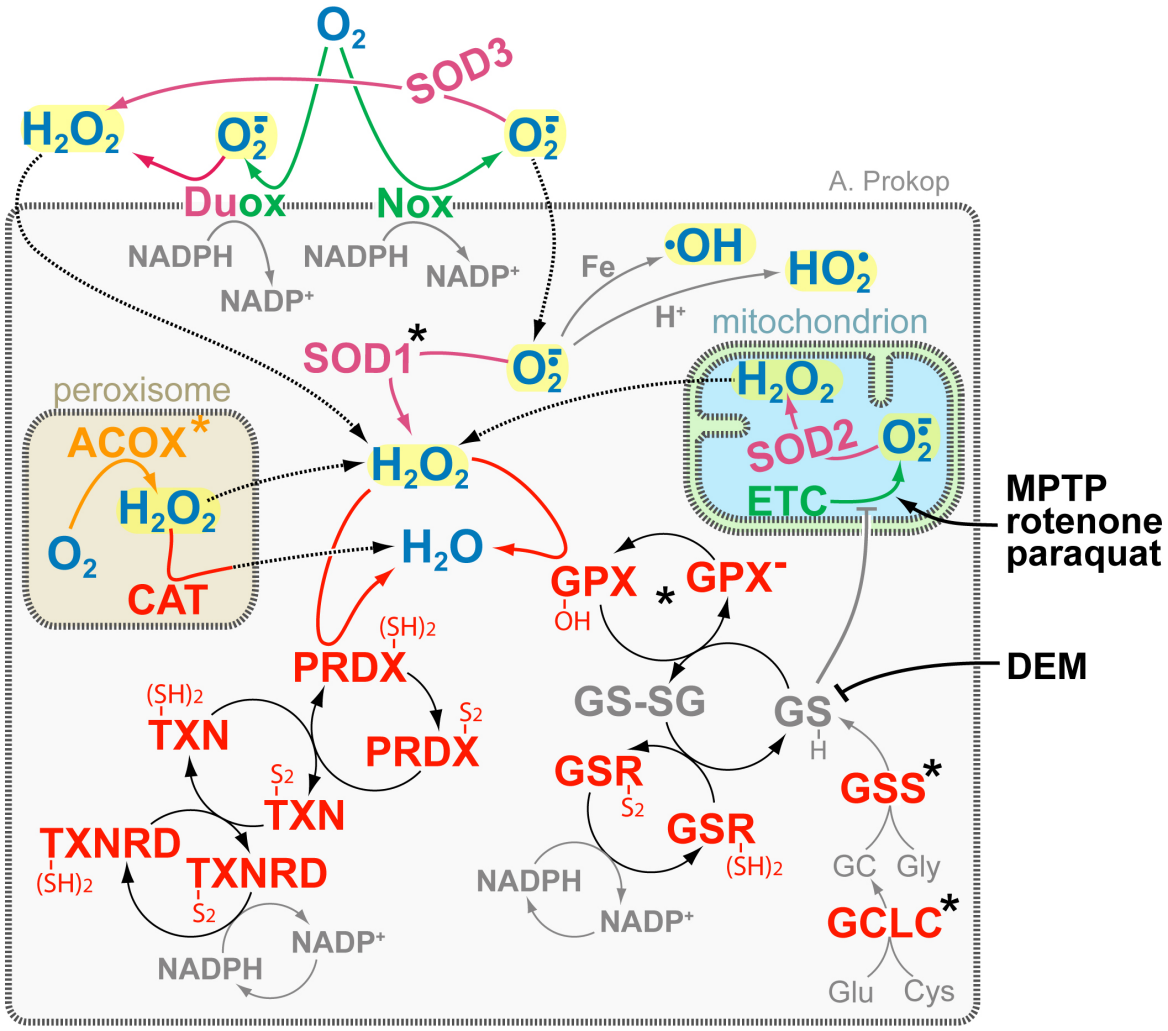
The complexity of ROS-generating and -regulating systems. Key ROS molecules (bold blue and highlighted in yellow) are hydrogen peroxide (H_2_O_2_) and superoxide (O_2_^·−^) with its derivatives hydroxyl (^•^OH) and perhydroxyl/hydroperoxyl (HO_2_^•^). Colour coding of enzymes: green, O_2_^·−^ producers; magenta, dismutases (reducing O_2_^·−^ to H_2_O_2_); orange, H_2_O_2_ producers; red, H_2_O_2_ reducers. Key redox agents (bold grey) are glutathione (GSH/GSSG) and NADPH/NADP^+^ (Willems et al., 2015), of which glutathione can also act directly (grey T-bar) on thiols of complex I of the electron transfer chain (ETC; Marí et al., 2020). Some ROS-inducing drugs are shown in black on the right: DEM blocks glutathione, whereas the toxic by-product of opioid production MPTP, the insecticide rotenone, and the herbicide paraquat induce ROS-by blocking complex I of the ETC (Szalárdy et al., 2015); the main grey box represents the cell, inset boxes peroxisomes and mitochondria as indicated, stippled arrows indicate diffusion, asterisks indicate proteins with OMIM-listed links to neural disorders; all abbreviations of gene names and their existing OMIM links are listed in Supplementary Table S3.

Peroxisomes perform various functions (Okumoto et al., 2020): (i) they execute α- and β-oxidation to break down fatty acids that cannot be processed by mitochondria; (ii) they carry out anabolism of brain-relevant products including glycerophospholipids and docosahexaenoic acid; and (iii) they contain catalase (‘Cat’ in Figure 4) as an enzyme that neutralises reactive oxygen species (ROS) derived from peroxisomal β-oxidation, but also mop up ROS from the cytoplasm.

The peroxisomal membrane appears to be permeable for many low-weight molecules, whereas others require enzymatic transport. For example, shuttle systems towards the cytoplasm replenish NAD^+^ pools required for β-oxidation, or molecules are exchanged with other organelles at membrane contact sites (Hua et al., 2017; Chen et al., 2020; Wanders et al., 2020): (a) peroxisomes pass on the short-chain acyl-CoA (i.e., products of their β-oxidation) to mitochondria for final catabolism; (b) ER passes on excess lipids to peroxisomes for β-oxidation, thus maintaining lipid homeostasis (‘13’ in Figure 1 >b/m); and (c) lysosomes pass on lipid degradation products for the same reason.

## Roles and regulations of the cytoskeleton in axons

The axonal cytoskeleton has two key functions: to grow and maintain axonal architecture and support or mediate intracellular transport and dynamics. The cytoskeleton comprises four different classes: neurofilaments and septins are filamentous polymers composed of variable non-polar subunits; F-actin and microtubules (MTs) represent polar filaments generated via head-to-tail polymerisation of their nucleotide-binding subunits, which are G-actin momomers and α/β-tubulin heterodimers, respectively. The latter two are regulated in comparable ways through actin- or MT-binding proteins which mediate nucleation of new filaments, their de−/polymerisation, cross-linkage, severing or post-translational modification (Prokop et al., 2013; Blanchoin et al., 2014; Brouhard and Rice, 2018).

F-actin filaments are ~5 nm thick. In axons, they form periodic cortical rings that are longitudinally interlinked by spectrins providing a regular spacing of ~190 nm between rings (‘Ac, S’ in Figure 1 >a/k; Xu K. et al., 2013; Costa and Sousa, 2021; Leterrier, 2021). This cortical actin-spectrin network forms a relatively rigid corset that gives axons their tubular appearance whilst providing spring-like properties to respond to pulling forces (Dubey et al., 2020; Chai et al., 2023). Stabilising these rings protects against axon fragmentation (Unsain et al., 2018; Wang et al., 2019). Furthermore, they might serve as a signalling hub or help to compartmentalise the axonal plasma membrane (Leterrier, 2021). Beside rings, axonal F-actin networks are essential for endo−/exocytosis, organelle shape dynamics and endosomal sorting (Gautreau et al., 2014; Kage et al., 2022). Dynamic F-actin networks in growth cones at neurite tips drive axonal morphogenesis including axon growth and branching (Lewis et al., 2013; Prokop et al., 2013; Kalil and Dent, 2014). Furthermore, actin patches or polarised actin networks at the axon initial segment were proposed to aid in selecting cargo for axonal transport (Watanabe et al., 2012; Balasanyan et al., 2017). Actin hotspots, trails or waves along axon shafts might mediate transport of actin (Flynn et al., 2009; Ganguly et al., 2015; Winans et al., 2016; Chakrabarty et al., 2019).

Microtubules (green lines in Figure 1) have a diameter of ~25 nm and lengths of up to several hundred micrometres; they are arranged in overlapping patterns into loose bundles that run uninterrupted all along axons (Prokop, 2020). These bundles form the highways for motor-driven cargo transport and movements, thus influencing virtually every aspect of axon biology (‘M’ in Figure 1 >b/g). The presence/absence of MT bundles closely correlates with the durability/destabilisation of axonal segments (Datar et al., 2019; Herwerth et al., 2022). Furthermore, MT bundles can spawn new MTs required for axonal growth or branching (Lewis et al., 2013; Kalil and Dent, 2014; Qu et al., 2019), and they contribute to signalling processes (Dent and Baas, 2014). This requires dynamic changes of MT networks driven by processes of nucleation, de−/polymerisation, repair, posttranslational modification and cross-linkage, all mediated by MT-binding proteins (‘Eb, X, Sp, 11, 18’ in Figure 1 >a + b/m + n and e/g; some details further below; Conde and Caceres, 2009; Hahn et al., 2019; Janke and Magiera, 2020). MTs were also reported to contain proteins in their lumen which might regulate MT stability; these include actin filaments, MAP 6 and chaperones (Cuveillier et al., 2020; Paul et al., 2020; Chakraborty et al., 2022; Tsuji and Dodding, 2022).

Microtubule bundles display quantitative and qualitative variations depending on neuron type, animal species and developmental stage. For example, the number of MT profiles per axonal cross-section can range from 1 to over 200,000, and the length of individual MTs from below 10 to hundreds of micrometres (Prokop, 2020). Furthermore, MTs can be composed of different tubulin isotypes or be subjected to different posttranslational modifications (jointly referred to as the ‘tubulin code’); these variations can have major impacts on MT properties and their interactions with MT-binding proteins including motor proteins (Janke and Magiera, 2020; Prokop, 2022).

Neurofilaments (pink lines in Figure 1) are ~10 nm thick polymers composed of ~60 nm long, non-polar subunits with multiple side arms, up to 60 nm long, of which the longer ones harbour numerous phosphorylation sites (Bott and Winckler, 2020). Neurofilaments are absent in arthropods, but prominent in larger diameter axons of other species including vertebrates (Prokop, 2020). In these axons, they form dynamic networks that run in parallel to MTs (Fenn et al., 2023), thus embedding MTs in a gel-like matrix and increasing axon diameters (Prokop, 2020). Besides neurofilament numbers further mechanisms reported to regulate axon diameters are the actin regulator ADD1 (= α-adducin) and the nucleocytoplasmic transport regulator IOP13 (= importin-13; Leite et al., 2016; Bin et al., 2023).

Septins are GTP-binding proteins which form hetero-hexa- or hetero-octamers that can assemble into filaments; they have diverse functions including membrane curvature sensing, or the regulation of actin and MT dynamics; in axons, they were demonstrated to regulate cargo transport, growth, branching and calcium homeostasis (Hu et al., 2012; Ageta-Ishihara et al., 2013; Spiliotis, 2018; Katz et al., 2019; Deb et al., 2020; Woods and Gladfelter, 2021; Nakos et al., 2022).

## The complex machinery of axonal transport in axons

Axonal transport is performed by MT-binding molecular motors (‘M’ in Figure 1 >b/g) which harbour motor domains that convert ATP hydrolysis-derived energy into mechanical force to move along MT lattices. Depending on motor class, they move on MTs via ‘processive’ walking in 4, 8, or 16 nm steps (Nishiyama et al., 2001; Sudhakar et al., 2021; Deguchi et al., 2022), via ‘non-processive’ hopping, or via surfing along MTs through ‘one-dimensional diffusion’ (Hunter and Allingham, 2020). Since MTs in axonal bundles predominantly point with their plus ends towards the distal tips of axons (Baas and Lin, 2011), this intrinsic polarity can be interpreted by motors to move either towards the cell body (retrograde) or to the axon tip (anterograde). Retrograde cargo transport is performed by large dynein-dynactin complexes; the processive subunit in these complexes are formed by homodimers of dynein heavy chains, each containing a C-terminal motor domain (Qiu et al., 2012; Schiavo et al., 2013; Reck-Peterson et al., 2018). Anterograde movements are performed by members of the kinesin superfamily comprising ~45 genes in vertebrates, grouped into 14 classes, or classified according to the position of their motor domains: N-, M- and C-KIFs carry the motor domain N-terminally, in the middle or C-terminally, respectively (Tanaka and Hirokawa, 2016). Kinesins involved in anterograde axonal cargo transport mostly belong to the type 1 (KIF5), 2 (KIF3 & 17) and 3 (KIF1 & 13) classes, but members of other classes may contribute (Hirokawa et al., 2010); all of them are processive N-Kifs with kinesin-1 and -3 having homo-dimeric and kinesin-2 heterodimeric motor units (Hammond et al., 2009; Muthukrishnan et al., 2009).

Consequently, only a handful of motor protein classes have to transport a vast range of very different cargoes including RNAs, lipids (vesicles, lipid droplets), proteins or protein complexes and even entire organelles (‘black motors’ in Figure 1; Hirokawa et al., 2010; Maday et al., 2014; Reck-Peterson et al., 2018); this complex logistical task is achieved through a modular system based on a plethora of adaptor/linker proteins or protein complexes that mediate the binding of different cargoes to the same motor, or of different motors to the same cargo (e.g., Bowman et al., 2000; Gindhart, 2006; Niwa et al., 2008; Hirokawa et al., 2010; Maday et al., 2014; Drerup et al., 2016; Brady and Morfini, 2017; Guedes-Dias and Holzbaur, 2019). In addition, messenger RNAs, proteins, lipid droplets or whole proteasomes have been reported to piggyback or hitchhike on transported vesicles or organelles (Otero et al., 2014; Kilwein and Welte, 2019; Liu et al., 2019; Minis et al., 2019; Vargas et al., 2022). Numerous challenges to our understanding of transport remain:

1. The different motor protein classes involved in axonal transport can act redundantly (Hirokawa et al., 2010) or even in co-operation (Zahavi et al., 2021), and some adaptors interact promiscuously with different motors. Due to this, unravelling motor-adaptor pairings requires painstaking systematic analyses (Hummel and Hoogenraad, 2021).
2. Transport can be fast or slow (40–80 vs. 0.2–10 mm/day, Twelvetrees, 2020), where slow transport is believed to be the consequence of low ‘duty-ratio’, which is the proportion of time that the cargo is pausing or moving (Roy, 2020). Slow transport may represent as short bouts of transport, for example driven by MT polymerisation (‘10’ in Figure 1 >a/n; Grigoriev et al., 2008; Pavez et al., 2019), or by infrequent short ‘hitchhiking’ events on fast moving cargo vesicles, as observed for synapsin (Tang et al., 2013) or neurofilaments (pink lines on ‘Cv’ in Figure 1 >b/g; Uchida et al., 2009; Wang and Brown, 2010).
3. There is a complex and little understood interdependence of kinesins and dynein: for example, to achieve relocation of these motors to their start positions or when regulating the directionality of transport (Uchida et al., 2009; Hendricks et al., 2010; Moughamian et al., 2013; Hancock, 2014; Twelvetrees et al., 2016), which might be regulated at the level of cargo adaptors (Canty et al., 2023).
4. Transport motors have additional functions which complicate their study: for example, they contribute to organelle morphogenesis (‘14, 22, 29’ in Figure 1 >b/n, c/k and d/n; Gautreau et al., 2014; Wang et al., 2015; Du et al., 2016; Özkan et al., 2021), or kinesin-1 performs MT sliding during axon growth (‘38’ in Figure 1 >f/n; Lu et al., 2015; Winding et al., 2016).
5. Further fundamental aspects of transport regulation remain little understood: (a) coordination of cargo loading/unloading at source/target sites (Kelliher et al., 2019); (b) mechanisms of MT track choice and transport duration (shown to involve phosphorylation of motors; alterations of MTs through posttranslational modification or decoration with MT-binding proteins Karasmanis et al., 2018; Monroy et al., 2020; Banerjee and Gunawardena, 2023; Genova et al. 2023; Li et al., 2023); (c) how transport can overcome physical barriers especially in narrow axons (Yu et al., 2017; Wang et al., 2020; images in Bowen et al., 2007); (d) Vesicular transport was shown to be fuelled by ‘on-board’ glycolysis (Figure 2; Zala et al., 2013; Hinckelmann et al., 2016; Yang et al., 2022a), but how ATP supply is ensured for the transport of other cargoes is not known (Chamberlain and Sheng, 2019).

## Roles and regulations of further cytoplasmic machineries

Besides the cytoskeletal and motor machineries, further cytoplasmic factors play important roles, for example to mediate processes of electrical and chemical signalling, metabolism, proteostasis and ROS homeostasis. Most of the processes have genetic links to neurodevelopmental and/or neurodegenerative disorders (Supplementary Table S1F). Here, we will focus on proteostasis and ROS homeostasis.

Proteostasis involves the balanced biogenesis, redistribution, repair and degradation of proteins (Cornejo et al., 2017; Hipp et al., 2019). As mentioned above, proteins can originate from neuronal cell bodies or glia cells and be transported into axons, but they may also be locally produced. Local production requires the presence of their mRNAs, ribonucleoproteins, ribosomes, translation factors, t-RNAs and amino acids, as well as the adequate chaperones. For cytoplasmic proteins, it has been convincingly demonstrated that local biogenesis takes place in axons as revealed by the presence of the above-listed components and the demonstration of on-site protein production (Spaulding and Burgess, 2017; Cioni et al., 2018; Kar et al., 2018; Vargas et al., 2022; Farberov et al., 2023; Pinho-Correia and Prokop, 2023; ‘32–37’ in Figure 1 >f/g-l).

There are some indications that also transmembrane or extracellular proteins are being locally produced in axons, although rough ER and canonical Golgi apparatus as the typical sites of biogenesis and distribution, appear to be restricted to neuronal cell bodies (Figure 1, top; Merianda et al., 2009; González et al., 2016; Cornejo et al., 2017; Luarte et al., 2018; Deng et al., 2021). This said, newly synthesised glutamate receptors in dendrites appear to bypass the Golgi on the way to the plasma membrane (Bowen et al., 2017). However, given the enormous importance of transmembrane or extracellular proteins in axons, more work is required to clarify whether they can be locally produced.

As already explained in a dedicated section above, the removal of protein aggregates and transmembrane proteins involves lysosome-dependent degradation (‘Ly’ in Figure 1 >c/j). In contrast, nuclear or cytoplasmic proteins are usually ubiquitinated and targeted for degradation to proteasomes which are present in axons (‘Pr’ in Figure 1 >f/l; Korhonen and Lindholm, 2004; Otero et al., 2014; Liu et al., 2019).

The generation of ROS is an important aspect of signalling processes driving neuronal and axonal development and function (Sena and Chandel, 2012; Hervera et al., 2018; Oswald et al., 2018a,b; Biswas et al., 2022). However, the ectopic presence of the wrong species or supra-threshold elevation of ROS levels can harm cells (oxidative stress), disrupting their cytoskeleton, lipids, organelle dynamics and functions with close links to neurodegeneration (Cadenas and Davies, 2000; Turrens, 2003; Wilson and Gonzalez-Billault, 2015; Wali et al., 2016; Liao P.C. et al., 2017; Liew et al., 2021; Yeung et al., 2021). As illustrated in Figure 4, typical ROS in cells comprise hyperoxide (H_2_O_2_) and superoxide (O_2_^·−^) which can react to generate further ROS. For example, O_2_^·−^ can undergo Fenton-like reactions with iron to generate the far more reactive hydroxyl radical (^•^OH; Zhao, 2019), or it can be protonated to form uncharged perhydroxyl/hydroperoxyl radicals (HO_2_^•^) able to penetrate and damage membranes (Panov, 2018). For signalling purposes, H_2_O_2_ and O_2_^·−^ can be generated by dedicated enzymes, such as membrane-associated DUOX or NOX (Brandes et al., 2014; Coelho de Faria and Fortunato, 2020; Figure 4). But ROS is also produced as by-product, for example of the mitochondrial electron transfer chain (‘ETC’ in Figure 4) or of enzymatic reactions driving β-oxidation (e.g., ‘ACOX’ in Figure 4; Camões et al., 2015), neurotransmitter or purine catalysis (e.g., MAOA/B, XDH; Liu et al., 2002; Okumoto et al., 2020; Wanders et al., 2020; Shields et al., 2021). Many of these ROS sources lie either outside the cell or in organelles separated from the cytoplasm by membranes which are little permeable for most ROS species; however, O_2_^·−^ and H_2_O_2_ were suggested to pass membranes through anion channels and specific aquaporins, respectively (Möller et al., 2019).

To maintain ROS homeostasis and protect against oxidative stress, specialised enzymes in cytoplasm and organelles convert O_2_^·−^ into H_2_O_2_ (magenta in Figure 4), and others reduce H_2_O_2_ to water (red in Figure 4). Some of these enzymes (e.g., ‘SOD2’, ‘CAT’ in Figure 4) are localised in organelles to buffer ROS at its production site, but they may also mop up ROS from the organelle’s environment, as is best described for peroxisomal CAT (Figure 4; Okumoto et al., 2020). Further protection from ROS in the cytoplasm or at the cell membrane is provided by SOD1 and SOD3 (reducing O_2_^·−^ to H_2_O_2_) as well as GPXs and PRDXs (neutralising H_2_O_2_), of which the latter two require associated cascade-like redox networks that ultimately use NADPH as reducing agents (Figure 4; Willems et al., 2015). One intermediate in these networks, glutathione (‘GSH’ in Figure 4), can enter mitochondria and act directly on thiols of the electron transfer chain (grey T-bar in Figure 4), thus quenching ROS at the site of its production (Marí et al., 2020). In contrast, over-abundance of oxidised glutathione (‘GS-SG’ in Figure 4) can affect mitochondrial dynamics *in vitro* and axons *in vivo* (Shutt et al., 2012; Smith et al., 2019). Due to its wide involvement in ROS protection, glutathione levels are often used as a biomarker for neuronal damage (Janáky et al., 2007).

## The house-of-cards principle: the dependency cycle of local axon homeostasis

**Figure 5:**
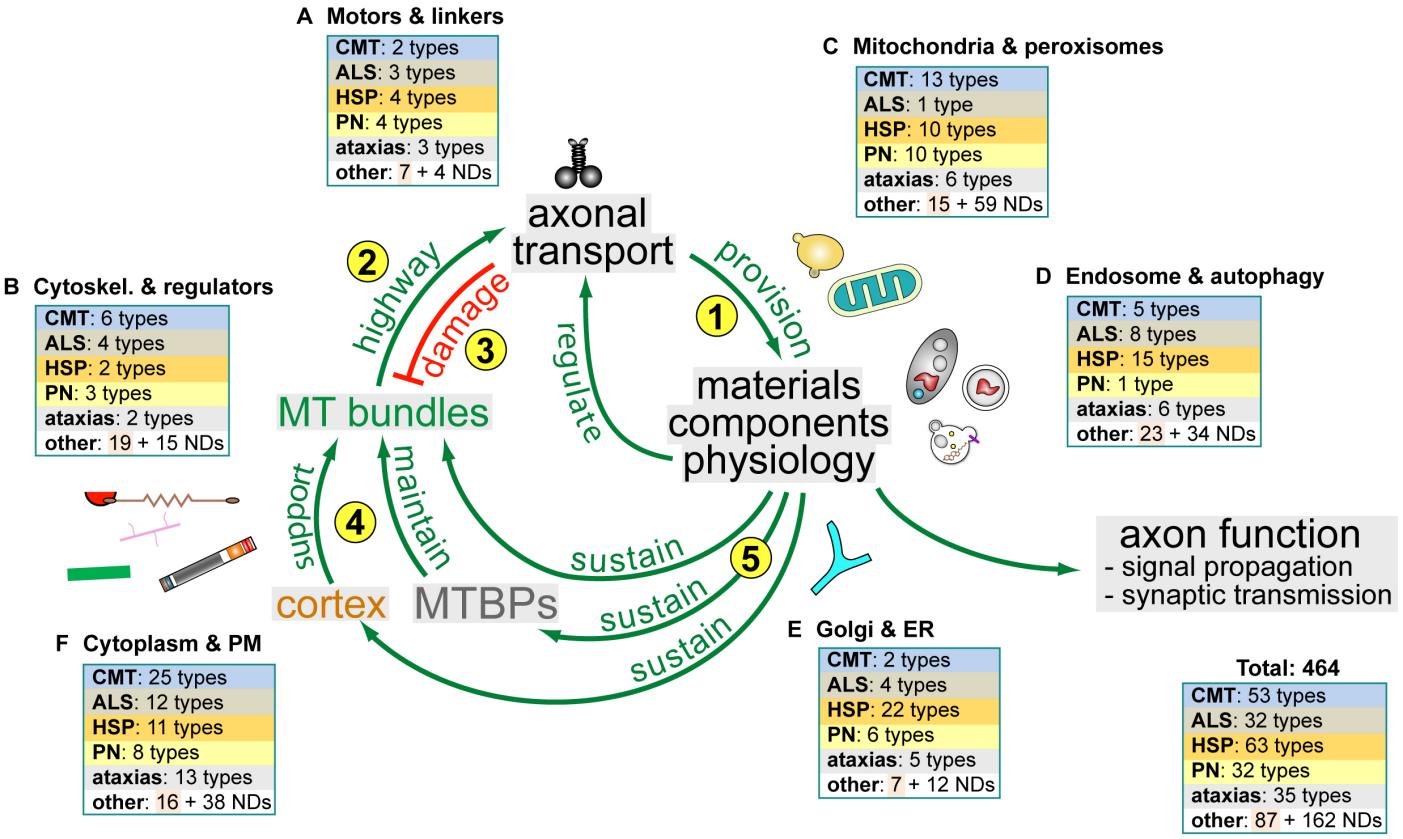
The dependency cycle of local axon homeostasis. Circular arrows in the centre represent the proposed circular dependencies: **(1)** motor protein-driven axonal transport supplies axons with materials and organelles required for axon function; **(2)** MT bundles are the key highways for axonal transport; **(3)** axonal transport damages MT bundles; **(4)** the cortical sleeve and the machinery composed of MT-binding and -regulating proteins (MTBPs) protect and maintain MT bundles long-term; **(5)** MT bundle maintenance depends on materials and physiology provided and upheld by axonal transport. Pictograms outside the circle reflect axonal components (as shown in Figure 1) to symbolise certain aspects of axonal cell biology involved in the respective processes. Boxes in the periphery reflect different axonal organelles or processes (labelled and named as in Supplementary Table S1) and illustrate how the hereditary disorders listed in Supplementary Table S1 map onto the cycle: disorders are classified as hereditary neurodegenerative (CMT, ALS, HSP, PN, ataxias and other) and neurodevelopmental (ND; all colour-coded as in Supplementary Table S1); the numbers in the boxes indicate how many genes linked to each class relate to the respective cell biological processes that make up the cycle.

As indicated by asterisks in the figures and as listed in Supplementary Table S1, almost every structure, organelle and process described above has well-established genetic links to neurodevelopmental or neurodegenerative disorders, many of which can be expected to affect axons. However, there is little indication that aberrations of specific cell biological processes would correlate with defined forms of axonopathy. For example, Riancho and co-authors stated in the context of ALS: ‘*although initial triggers may differ among patients, the final motor neurons’ degeneration mechanisms are similar in most patients once the disease is fully established*’ (Riancho et al., 2019). This statement is corroborated by our data mining exercise with the ‘Online Mendelian Inheritance in Man’ (OMIM) database, showing that CMT, ALS, HSP, peripheral neuropathies or ataxias can all be caused by mutations in a spectrum of genes that relate to virtually every cell biological process described in this article (colour-coded in Figure 5 and Supplementary Table S1). Like removing a randomly chosen single card can lead to the collapse of an entire card house, it seems to be of little relevance through what mechanism axonal homeostasis is corrupted and axonal deterioration triggered. It seems more important which neuron type is most vulnerable to a certain genetic defect, which will then determine the class of axonopathy.

A potential explanation for this card house phenomenon is provided by the fact that virtually all axonal structures, organelles and processes are intimately connected to each other: (1) membrane contact sites are formed between most or even all organelles and also with the plasma membrane; (2) Ca^2+^ and ROS levels act as second messengers to orchestrate systemic changes across cell biological processes, and they are regulated through joint networks of factors localising to organelles, in the cytoplasm and at the plasma membrane; (3) also lipidogenesis and metabolic processes involve enzymes in all these locations and require contact sites or transporters to exchange products between them; and (4) virtually all organelles and processes depend on the cytoskeleton and transport machinery to perform their dynamics and achieve their proper distributions.

**Figure 6:**
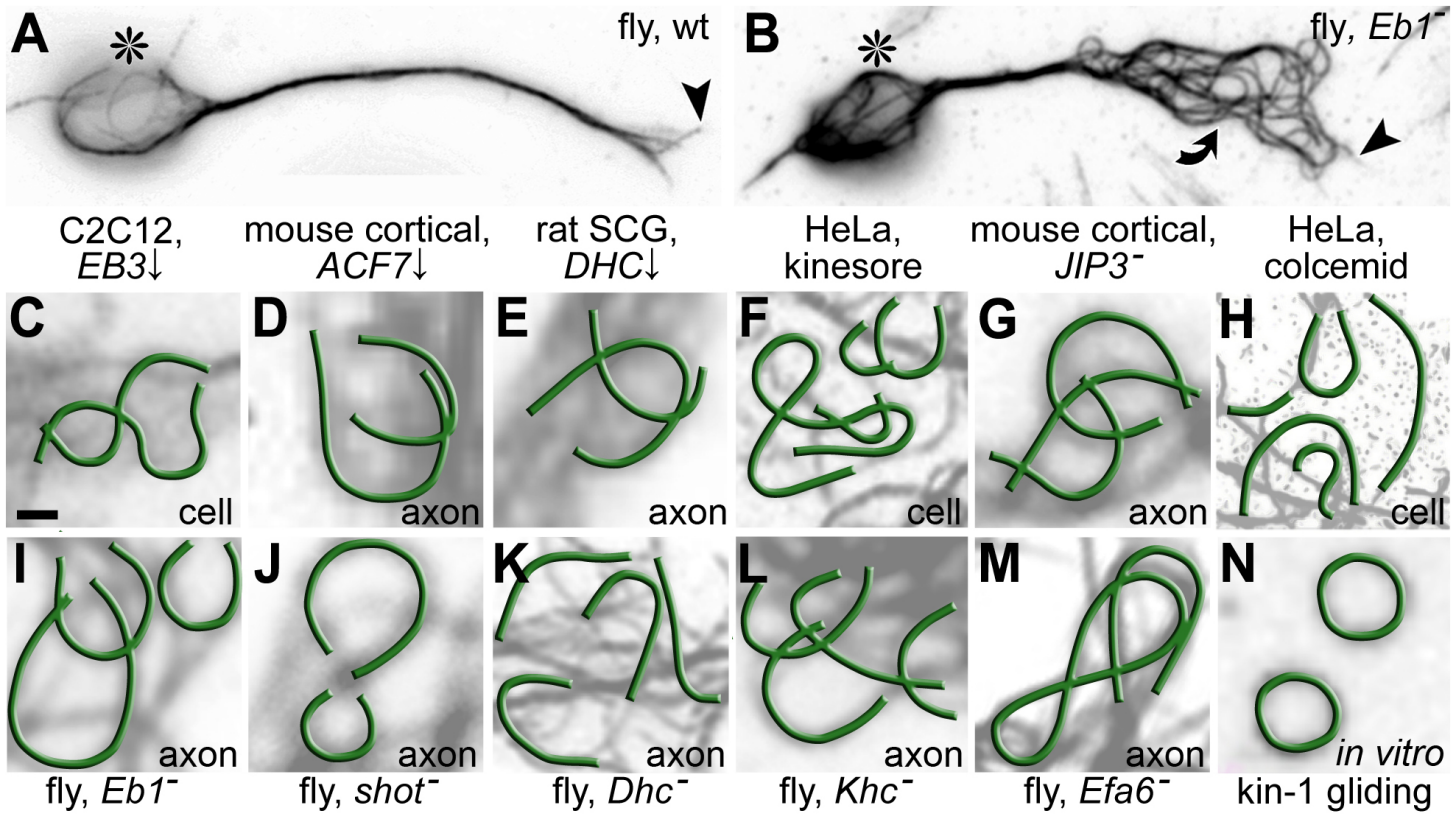
Microtubule (MT) curling as a potential pathological phenomenon in axons and non-neuronal cells. **(A,B)** Primary *Drosophila* neurons cultured for 1 day *in vitro* either from wild-type or *Eb1* mutant embryos (asterisks, cell bodies; arrow heads, axon tips; curved arrow, area of MT curling). **(C–N)** Published examples at same scale of disorganised MT curling taken from axons or non-neuronal cells (as indicated bottom right): **(C)** Mouse C2C12 myoblast cell line depleted of the MT plus end scaffold MAPRE3 (=EB3; modified from Figure 3A in Straube and Merdes, 2007); **(D)** Mouse primary cortical neurons depleted of the spectraplakins MACF1 (modified from Figure 2E in Sánchez-Soriano et al., 2009); **(E)** Rat primary superior cervical ganglia neurons depleted of the motor complex component dynein heavy chain (DHC; modified from Figure 7I in Ahmad et al., 2006); **(F)** HeLa cells treated with 50 μM of the kinesin-1-activating drug kinesore (modified from Figure 2A in Randall et al., 2017); **(G)** Primary cortical mouse neurons upon MAPK8IP3 (= JIP3) knock-out (modified from Figure 2E in Rafiq et al., 2022); **(H)** HeLa cells treated with 80 nM of the cancer therapy drug colcemid (modified from Figure 2A in Rozario et al., 2021); **(I–M)** *Drosophila* primary neurons with genetic loss of function for the MT plus end scaffold Eb1, the spectraplakin Shot, the MT motor proteins Dynein heavy chain (Dhc) and Kinesin heavy chain (Khc) as well as the cortical collapse factor Efa6 (modified from Hahn et al., 2019; Liew et al., 2021); **(N)** A kinesin-1-containing *in vitro* gliding assay containing 5 mg/mL MTs (3% rhodamine-labelled; modified from Figure 2B in Liu et al., 2011). Scale bar in **(A)** represents 1 μm in all images.

As a cautious attempt to bring some order into this complex interdependent network of axonal biology, the ‘dependency cycle of local axon homeostasis’ model has been proposed which consists of 5 steps (yellow numbers in Figure 5; Prokop, 2021):

1. Motor-driven axonal transport (‘M’ in Figure 1 >b/g) is essential to provide the materials and organelles required for axonal function and maintenance.
2. The highways for this transport are the loose but continuous bundles of MTs that run all along axons (green lines in Figure 1).
3. The wear-and-tear and mechanical load of this transport (vibration lines in Figure 1) cause structural damage to MT bundles including pathological curling (Figure 6).
4. MT-binding proteins (‘MTBPs’ in Figure 5) counter-balance this damage through active MT bundle maintenance; further structural support is provided by the cortical actin-spectrin corset (‘Ac’ and ‘S’ in Figure 1; ‘>a/k’ Qu et al., 2017; Dubey et al., 2020).
5. Finally, MT-bundle maintenance depends on biological materials and physiological conditions provided through axonal transport—and this last step closes a circular arrangement of dependencies where interruption in any position of the cycle will have knock-on effects on all the others.

In the following, we will summarise current knowledge of how organelle dysfunction may affect axonal homeostasis and whether the dependency cycle is a suitable model to absorb such processes into a meaningful concept explaining axonopathies.

## Links of axonal transport to axonal disorders

The availability, proper distribution and/or dynamics of components relating to virtually every axonal cell biological process depend on motor-mediated transport or force generation (black motors in Figure 1), rendering axonal homeostasis highly vulnerable to the dysfunction of motor and cargo adaptor proteins (‘1’ and ‘5’ in Figure 5). Accordingly, function-impairing mutations of KIF1 (kinesin-3), KIF5 (kinesin-1), dynein and several of their cargo adaptors have well-established OMIM links to axonopathies (Supplementary Table S1A, ‘Motors and linkers’ in Figure 5; Kawaguchi, 2013; Schiavo et al., 2013; Appert-Rolland et al., 2015; Brady and Morfini, 2017; Prior et al., 2017; Guedes-Dias and Holzbaur, 2019; Sleigh et al., 2019; Guo et al., 2020).

Many links relate to failed organelle transport which will be discussed in the organelle section below. Another link relates to failed transport of the NAD^+^-producing enzyme NMNAT2 which leads to the accumulation of its substrate NMN which, in turn, triggers axonal degeneration mediated by the NAD^+^-catabolising enzyme SARM1 (Gilley et al., 2017; Hopkins et al., 2021; Li et al., 2022; Yang et al., 2022a; Loreto et al., 2023; Figure 2C).

A secondary consequence of failed transport can be the occurrence of pathological cargo aggregations, which can then have knock-on effects through indiscriminate roadblocks for other transport processes; in the same way, also aggregations caused by transport-independent means can lead to random roadblocks (Collard et al., 1995; Lai et al., 2018; Fumagalli et al., 2021).

A further important factor is the activity status of the transport motors KIF5, KIF1 and dynein: all these motors auto-inactivate and detach from MTs when bare of cargo (Hammond et al., 2009; Kaan et al., 2011; Reck-Peterson et al., 2018). This auto-inactivation usually requires co-factors: for example, KIF1A requires the KIFBP adaptor (Kevenaar et al., 2016; Cong et al., 2021; Solon et al., 2021), and KIF5 requires the KLC adaptor (Verhey et al., 1998; Bowman et al., 2000; Koushika, 2008; Verhey and Hammond, 2009; Wong and Rice, 2010; Yip et al., 2016; Randall et al., 2017; Paul et al., 2020; Tan et al., 2023). The dependency cycle of local axon homeostasis would predict that hyper-activation of motors through failed auto-inhibition should cause MT bundle damage (‘3’ in Figure 5), and this may be a reasonable explanation for a number of reports: (i) Hyperactivation of *Drosophila* kinesin-1 is a lethal condition accompanied by severe MT curling (Brendza et al., 1999; Kelliher et al., 2018; Liew et al., 2021), and such curling is similarly observed upon kinesin-1 activation in non-neuronal cells (Randall et al., 2017; Figure 6). (ii) Hyperactivating kinesin mutations in humans have been linked to axonopathies (KIF5A^∆Exon27^ causes ALS; Nicolas et al., 2018; Saez-Atienzar et al., 2020; Naruse et al., 2021; Baron et al., 2022; KIF1A^V8M^, KIF1A^A255V^ and KIF1A^R350G^ cause HSP/SPG; Chiba et al., 2019; Gabrych et al., 2019). (iii) KIFBP mutations are disruptive to axonal MT bundles and cause Goldberg-Shprintzen syndrome (‘GOSHS’ in Supplementary Table S1A; Lyons et al., 2008; Hirst et al., 2017; Chang et al., 2019). (iv) KLC mutations are linked to HSP/SPG and SPOAN (Supplementary Table S1A; Bayrakli et al., 2015; Melo et al., 2015; Fu et al., 2021; Gümüşderelioğlu et al., 2023) and to excessive axon branching in zebrafish (Haynes et al., 2022).

## Links of the cytoskeleton and its regulators to axonal disorders

Neurofilaments are highly abundant in many axons, but their loss in human, mouse or quail is not a lethal condition (Prokop, 2020; ‘2’ and ‘4’ in Figure 5). Their absence was even shown to be beneficial in tau-transgenic mouse models of neurodegeneration (Williamson et al., 1998; Ishihara et al., 2001). Instead, pathological neurofilament aggregation eventually leading to axonopathies can be caused by dominant mutations in neurofilament genes themselves (e.g., ‘CMT2B1, CMT2E, CMT1F, CMT1F, ALS1’ in Supplementary Table S1B) or through mutation of potential neurofilament regulators (e.g., ‘GAN, CMT2F’ in Supplementary Table S1B; Perrot and Eyer, 2009; Hentati et al., 2013; Khalil et al., 2018). Such defects may cause derailed neurofilament transport or affect filament assembly or cross-linkage.

Genes encoding actin, actin regulators (e.g., formin, profilin) or cortex components (e.g., adducin, β-spectrin and ankyrin) have links to neurodegenerative disorders, but through routes that are little understood. For example, mutations of the actin-binding protein PFN1 can cause ALS, but the underlying mechanisms seem to involve unconventional roles in MT regulation (Henty-Ridilla et al., 2017; Pinto-Costa and Sousa, 2020). Loss of cortical actin networks including spectrins, adducins and ankyrins, render axons and axonal MT bundles vulnerable to mechanical damage (Hammarlund et al., 2007; Krieg et al., 2017). They can affect the AIS, nodes of Ranvier, axon growth and ion channel distributions (Liu and Rasband, 2019; Lorenzo et al., 2023), cause changes in axon diameters (Leite et al., 2016; Costa et al., 2020), and negatively impacts MT polymerisation (potentially mediated by mechano-sensing of decreased axonal surface rigidity; Heidemann and Buxbaum, 1990; Qu et al., 2017). Overall, there is a reasonable case to suggest that the axonal cortex protects and helps to maintain axons long-term (‘4’ in Figure 5).

Dysfunctional MT bundle regulation can lead to two major phenotypes. Firstly, it may cause aberrant axonal growth and branching as well as axon retraction, reflecting key roles of MTs in implementing and maintaining axonal morphology (Hahn et al., 2019; Qu et al., 2019). Secondly, aberrant MT bundle regulation may cause axonal transport deficits due to gaps or polarity flaws in MT bundles, chaotic curling of MTs, or disturbed MT-motor interactions (Yamasaki et al., 1991; Bernier and Kothary, 1998; Dalpe et al., 1998; Tarrade et al., 2006; Fiala et al., 2007; Adalbert et al., 2009; Tang-Schomer et al., 2012; Fassier et al., 2013; Niwa et al., 2013; Denton et al., 2014; Havlicek et al., 2014; Hsu et al., 2014; Sorbara et al., 2014; Yin et al., 2016; Magiera et al., 2018).

On the one hand, the causes for axonal MT bundle defects can lie in mutations of tubulin genes, close to a hundred of which have been linked to neural disorders (‘The Tubulin Mutation Collection’: tubulinmutations.bio.uci.edu/alpha-tubdb.html; Supplementary Table S1B). These mutations may interfere with tubulin biogenesis, MT nucleation or polymerisation, or the interaction of MTs with MT-binding proteins (Kumar et al., 2010; Poirier et al., 2010; Tischfield et al., 2011; Poirier et al., 2013; Bahi-Buisson et al., 2014; Fallet-Bianco et al., 2014; Smith et al., 2014; Aiken et al., 2017; Gonçalves et al., 2018; Romaniello et al., 2018; Pham and Morrissette, 2019; Fourel and Boscheron, 2020; Hausrat et al., 2022; Prokop, 2022).

On the other hand, MT bundle defects can be caused by dysfunctional MT-binding and -regulating proteins (‘MTBPs’ and ‘4’ in Figure 5; Hahn et al., 2019). For example, MT-crosslinking proteins (‘18’ in Figure 1>e/g) have long been suggested to be important for MT bundle architecture, but the workings and relevance of bundle cross-linkers remains little understood (Bodakuntla et al., 2019; Prokop, 2020; Guha et al., 2021). In contrast, substantial evidence has been provided for the importance of regulated MT polymerisation as a mechanisms of MT turnover: for example, the extension of polymerising MTs is guided by spectraplakins along the axonal cortex into parallel bundles, as shown both in mouse and *Drosophila* neurons (‘11’ in Figure 1>b/n; Sánchez-Soriano et al., 2009; Alves-Silva et al., 2012; Voelzmann et al., 2017; Hahn et al., 2021; Qu et al., 2022); cortical collapse factors act as quality control factors selectively eliminating polymerising MTs, which escape the bundled arrangements (Qu et al., 2019). The typical phenotype when such guidance and elimination mechanisms fail, is the transition of bundled arrangements into disordered areas of criss-crossing MTs forming curls with diameters in the lower micrometre range – and such curling is an increasingly recognised pathological phenomenon in neuronal axons but also non-neuronal cells (Figure 6).

Observations from *in vitro* MT gliding assays suggest that MT curling might be induced by motor forces (Lam et al., 2016; Hahn et al., 2019; Figure 6N, ‘3’ in Figure 5). Once curling is induced, it has been suggested to persist as an energetically favoured tubulin conformation (Ziebert et al., 2015), further stabilised through MT-binding proteins (Pearce et al., 2018; Peet et al., 2018). If this motor-induced tendency to curl is indeed counter-balanced by MTBP-dependent maintenance mechanisms (‘4’ in Figure 5), then disturbances of this homeostatic balance in favour of the motor-induced damage, should lead to a common phenotype. This would explain why the same MT curling is observed upon hyper-activation of motors in axons and non-neuronal cells (previous section; ‘3’ in Figure 5), but also when axonal MT guidance or cortical MT elimination mechanisms fail (previous paragraph; ‘4’ in Figure 5). A further cause for curling may be structural damage of MTs through motor-induced wear-and-tear (Dumont et al., 2015; VanDelinder et al., 2016; Andreu-Carbó et al., 2021; Budaitis et al., 2021; Wu et al., 2022). Usually, damage is responded to by MT repair mechanisms (Aumeier et al., 2016; Aher et al., 2020; Triclin et al., 2021), but a temporarily weakened MT lattice may be more vulnerable to curl induction.

## Links of organelles to axonal disorders

The enormous importance of the various organelles described in the first part would suggest that failure of any of the many functions they perform will impact on axonal homeostasis, hence maintenance (Supplementary Tables S1C-E; ‘5’ in Figure 5).

For example, autopsies of neurodegeneration patients often reveal aberrations of mitochondria (Pathak et al., 2013). Mitochondrial pathologies can be caused by dysfunction of OXPHOS or their metabolism, or by aberration of their biogenesis including mitochondrial DNA replication and translation (Antonicka et al., 2010; Natera-Naranjo et al., 2012; Xu W. et al., 2013; Tucci et al., 2014; Alonzo et al., 2018), or of their degradation and fission/fusion dynamics (Baloh et al., 2007; Misko et al., 2012; Rizzo et al., 2016; Rocha et al., 2018; Longo et al., 2020). For example, mutations in almost all nuclear genes for mitochondrial aminoacyl-transfer RNA synthetases and mitochondrial genes for transfer RNAs have links to neural disorders (Sissler et al., 2017; misynpat.org/misynpat/DiseasesOverview.rvt; Supplementary Table S1C). Mitochondrial dysfunction can also be caused by Ca^2+^ dysregulation, for example through mutations in mitochondrial Ca^2+^-regulating proteins (Liao Y. et al., 2017; Jung et al., 2020), in response to oxidative stress (Barrett et al., 2014), or through aberrant communication with the ER (Villegas et al., 2014). Sustained Ca^2+^ stress may trigger opening of the high-conductance mPTP believed to be formed by dimers of the F_0_F_1_ ATP synthase (Figure 3C; Bonora et al., 2022). This can lead to mitochondrial decay and even cause cellular necrosis or apoptosis, of which apoptosis involves the release of death factors including cytochrome C (‘CYCS’ in Figure 3C) through BAX/BAK pores in the outer mitochondrial membrane (Figure 3C; Zhang et al., 2017). If locally restricted, apoptosis can act as a mechanism of axon degeneration (Smith and Gallo, 2018; Geden et al., 2019).

Almost all mitochondria-related axonopathy-associated mutations ultimately give rise to heightened ROS, which can then have secondary enhancing effects through the cessation of mitochondrial trafficking leading to improper positioning of mitochondria along axons. Improving mitochondrial trafficking might therefore be a promising target for therapies addressing neurodegenerative conditions or ageing (Mattedi and Vagnoni, 2019). This notion is further supported by recent reports that deficient transport of mitochondria upon loss of Khc, Milton or Miro in fly primary neurons causes severe MT curling (Figure 6L); this phenotype was mediated by harmful ROS induction (Liew et al., 2021). In the context of the dependency cycle of local axon homeostasis, this example suggests that axonal transport of mitochondria is important to sustain physiological homeostasis which, in turn, is important for MT bundle maintenance, thus establishing a circular relationship (‘5’ in Figure 5).

In support of this circular relationship, also defective lysosome transport caused by knock-down of JIP3 (= MAPK8IP3) in mouse primary neurons caused MT curling, which was reported to be correlated with hyperphosphorylation of tau expected to trigger tau detachment from MTs (Rafiq et al., 2022). This agrees with findings in axons of fly and *Xenopus* neurons showing that loss of tau causes MT curling (Hahn et al., 2021). Also transport aberrations of other components of the endomembrane system relate to axonal pathologies. For example, mutations of the autophagy adaptors OPTN, UBQLN2, SQSTM1 and TBK1 (a kinase for OPTN) all cause severe deficits in autophagy and link to different forms of ALS and FTD (Supplementary Table S1D; Deng et al., 2011; Fecto et al., 2011; Burk and Pasterkamp, 2019); mutations of the ESCRTIII complex component CHMP2B cause FTDALS7/FTD3 (Skibinski et al., 2005; Clayton et al., 2015).

A different mechanistic route to inherited endomembrane-related pathologies is dysfunctional metabolism through the depletion of enzymes or trans-membrane transporters. For example, Krabbe disease is caused by mutations in the lysosomal enzyme galactosylceramidase (‘GALC’ in Supplementary Table S1D) resulting in pathological accumulation of the cytotoxin psychosine (Kreher et al., 2022). Mutations of the cation channel mucolipin 1 (=TRPML1; ‘MCOLN1’ in Supplementary Table S1D) cause mucolipidosis IV through over-acidification, thus blocking pH-sensitive lysosomal metabolism (Soyombo et al., 2006). Mutations in mucolipin also cause oxidative stress due to its role as a ROS-sensing and -responding factor (Soyombo et al., 2006; Santoni et al., 2020).

Dysfunctional enzymes may not only affect metabolism but also endomembrane trafficking. For example, when blocking cathepsin activity or reducing acidification in late endosomes, endo-lysosomal trafficking was inhibited leading to the accumulation in dystrophic axonal swellings, whereas dynamics of mitochondria were unaffected (Lee et al., 2011). Similarly, lysosomal trafficking was affected by loss of the lipid transporter NPC1 causative in Niemann-Pick disease (Roney et al., 2021), or by mutation of β-glucosidase linked to Gaucher disease (‘GBA’ in Supplementary Table S1) and PARK9 (Sidransky et al., 2009; Zigdon et al., 2017; Berge-Seidl and Toft, 2020).

Trafficking defects can also be triggered through independent causes: for example, extracellular amyloid plaques in Alzheimer’s pathology cause lysosome-filled swellings in adjacent axons by blocking their axonal transport (Drerup and Nechiporuk, 2013; Gowrishankar et al., 2015, 2017, 2021). Vice versa, blocking lysosome traffic via loss of Jip3 caused an over-production of Aβ_42_ (the main component of amyloid plaques), suggesting a detrimental feedback loop between plaque growth and blocked lysosomal traffic (Gowrishankar et al., 2017, 2021).

Also ER-related genes have strong links to axonopathies which, for unknown reasons, have a prevalence for HSP/SPG that primarily affects upper motorneurons (~40% or ER-linked diseases listed in Supplementary Table S1E). Upper/lower motorneurons and sensory neurons have the longest axons in our bodies, potentially rendering them more vulnerable to having pathological gaps in their continuous ER networks. Since upper motorneurons tend to be smaller in diameter than their counterparts in peripheral nerves (Prokop, 2020), this might further increase this vulnerability because they would be expected to contain fewer longitudinal ER tubules, meaning there is less compensatory capacity if some of these tubules are interrupted. In agreement with this notion, mutations in various factors involved in ER morphogenesis (i.e., REEP, ATL or RTN proteins) can cause aberrant ER morphology (Orso et al., 2009; Park et al., 2010; Rao et al., 2016; Yalçin et al., 2017; Zamponi et al., 2022; Zhu et al., 2022) and have links to HSP/SPG (but also peripheral neuropathies; Supplementary Table S1).

A potential link from aberrant ER morphology to disturbed axon homeostasis might be deficient Ca^2+^ metabolism and its known impact on axonal maintenance (Villegas et al., 2014). Also increased Ca^2+^ release from the ER can be a mediator of axon decay, as is the case when the axonal death factor SARM1 is activated (Coleman and Höke, 2020; Li et al., 2022; green inset box in Figure 2). Since rough ER as an important site of protein production appears sparse in mature axons it appears questionable whether axonopathies can arise from ER stress caused by intra-tubular aggregation of aberrant proteins (Scheper and Hoozemans, 2015). This said, key sensors of the unfolded protein response machinery including ERN1 (= IRE1α), EIF2AK3 (= PERK) and ATF6 are of benefit during axon regeneration (Oñate et al., 2016; Ohtake et al., 2018; Adams et al., 2019)—i.e., a condition with more need for localised protein synthesis and export than during the maintenance of healthy axons.

## Conclusion

Most axons survive for as long as we live through the homeostasis of a vast network of closely interwoven processes involving all components of axonal cell biology. Derailing this homeostasis at any position can trigger axonopathies. The dependency cycle of local axon homeostasis can help to make sense of the functional links between the different aspects of axonal cell biology and provide new opportunities to integrate and align different areas of axon research. Here we focussed on inherited neurodegenerative disorders because they have defined genetic lesions in specific organelles or cellular processes, hence break the cycle in identifiable positions (although this is less true for mutations that cause dominant gain of function phenotypes last column in Supplementary Table S1; Kim et al., 2020).

As argued above, different axonopathy classes cannot be defined as ‘mitochondrial’, ‘lysosomal storage’, ‘cytoskeletal’ or ‘transport’ diseases (Figure 5). Instead, each of these axonopathy classes links to a range of genes relating to a broad spectrum of cell biological contexts. It seems less relevant in which position the dependency cycle is broken, since it will eventually lead to a similar outcome of axon decay. It seems therefore important to understand why mutations in different genes involved in the homeostasis of the same organelle, say mitochondria, can differentially affect different neuron types (e.g., motorneurons, sensory neurons or neurons in the brain), thus leading to different classes of axonopathies. We need to understand the specific biology and vulnerabilities of these neurons to learn how to slow down progression of a certain axonopathy class. In times of personalised medicine, this would also help to design potential therapeutic strategies that have the widest possible efficacy. This said, the dependency cycle also seems to support the idea that certain aspects of axonal cell biology might be particularly suitable for therapies to stabilise axons against decay. MT bundles certainly are good candidates to this end (Alle et al., 2023).

Finally, the dependency cycle may also help to gain a better understanding of acquired axonopathies (e.g., caused by ageing, trauma, poisoning or chemotherapy, metabolic diseases, demyelination or pathogen-induced inflammation; Salvadores et al., 2017; Song et al., 2022), which may often affect a range of cell biological processes in parallel, but will eventually break the same cycle as the more focussed genetic lesions.

## Author contributions

GS, SS, CJO’K, and AP contributed to the writing, figures, and editing of the final text version. All authors contributed to the article and approved the submitted version.

## Funding

Work underpinning this article was made possible through support by the BBSRC to AP (BB/I002448/1, BB/P020151/1, BB/L000717/1, and BB/M007553/1), the MRC (MC_PC_16030/1) and Leverhulme Trust (RPG-2020-369) to GS, the Alzheimer’s Society United Kingdom (AS-PG-2013-005) and MRC (MR/M013596/1) to SS, and from the BBSRC (BB/L021706/1), MRC (MR/S011226/1) and Spastic Paraplegia Foundation to CJO’K.

## Conflict of interest

The authors declare that the research was conducted in the absence of any commercial or financial relationships that could be construed as a potential conflict of interest.

## Publisher’s note

All claims expressed in this article are solely those of the authors and do not necessarily represent those of their affiliated organizations, or those of the publisher, the editors and the reviewers. Any product that may be evaluated in this article, or claim that may be made by its manufacturer, is not guaranteed or endorsed by the publisher.
