# Supplementary material for: How neurons maintain their axons long-term: an integrated view of axon biology and pathology
Source: Front Neurosci. 2023 Jul 26;17:1236815. doi: 10.3389/fnins.2023.1236815 (PMC10410161; doi:10.3389/fnins.2023.1236815)
Supplement: Supplementary file 1 [file Table_1.pdf]

developmental | CMT | ALS | HSP | ataxias | peripheral neurop.

Gene name	OMIM	function	Disease	Ref.	Genet. class
**A) Motors & linkers**					
DYNC1H1	600112	retrograde transport	CMT2O	614228	AD
			MRD13	614563	AD
			SMALED1	158600	AD
DCTN1	601143	retrograde transport	ALS1	105400	AD/R
			HMN7B	607641	AD
			Perry Syndr.	168605	AD
KIF1A	601255	anterograde transport	SPG30	610357	AD/R
			HSN2C	614213	AR
			NESCAVS (PN, ataxia)	614255	AD
			HSP	[Ref.1]	?
			ataxias		?
			KAND	[Ref.2]	?
KIF1B	605995	anterograde transport	CMT2A1	118210	AD
KIF1C	603060	anterograde transport	SPAX2	611302	AR
KIF5A	602821	anterograde transport	SPG10	604187	AD
			ALS25	617921	AD
			CMT2	[Ref. 3]	?
KLC2	611729	KIF5-associated linker	SPOAN (PN)	609541	AR
KLC4	-		SPG	[Ref.8]	AR
KIF15	617569	spindle/Golgi/vesicle transport	BRDCS2	619981	AR
MAPK8IP3 / JIP3	605431	transport linker	NEDBA	618443	AD
KIFBP	609367	KIF1 linker (mitochondr. Transp.)	GOSHS	609460	AR
TRAK1	608112	mitochondrial transport linker	DEE68	618201	AR
PAFAH1B1 / LIS1	601545	dynein regulator	LIS1	607432	AD
NDE1	609449	mitochondrial transport	LIS4	614019	AR
ANXA11	602572	RNA transport linker	ALS23	617839	AD
			IBMWMA	619733	AD
**B) Cytoskeleton & its regulators**					
MAPT	157140	MT lattice-binding	FTD	600274	AD
			Pick disease	172700	AD
			PSNP1	157140	AD
			PD syndrome	260540	AR
MAP1B	157129		PVNH9	618918	AD
EML1 / EMAP	602033		BH	600348	AR
KIF21A	608283	cortical MT collapse factor	CFEOM1	135700	AD
KIF2A	602591	MT depolymerisation	CDCBM3	615411	AD
SPAST	604277	MT severing	SPG4	182601	AD
DST	113810	spectraplakins, actin-MT-neurofilament cross-linkage	HSAN6	614653	AR
MACF1	608271		LIS9	618325	AD
KATNB1	602703	MT severing	LIS6	616212	AR
CCT5	610150	chaperonin for actin/tubulin	HSN SPG	256840	AR

TBCD	604649	tubulin chaperone	PEBAT	617193; [Ref.7]	AR
TBCE	604934		PEAMO	617207	AR
DCX	300121	MT polymerisation dynamics	LISX1	300067	XL
ELP1	603722	α-tubulin acetylation?	HSAN3	223900	AR
TUBA4A	191110	α-tubulin	ALS22	616208	AD
TUBA1A	602529		LIS3	611603	AD
TUBB	191130	β-tubulin	CDCBM6	615771	AD
TUBB2A	615101		CDCBM5	615763	AD
TUBB2B	610031		CDCBM7	612850	AD
TUBB3	602661		CDCBM1	614039	AD
			CFEOM3A	600638	AD
TUBB4A	602662		HLD6	612438	AD
LMNA	150330	nuclear intermediate filament	CMT2B1	605588	AR
NEFL	162280	neurofilament protein	CMT2E	607684	AD
			CMT1F	607734	AD/R
			CMTDIG	617882	AD
NEFH	162230	neurofilament protein	CMT2CC	616924	AD
			ALS1	105400	AD/R
PRPH	170710	Class-III neurofilament	ALS1	105400	AD/R
DES	125660	type III intermediate filament	SCPNK	181400	AD
ADD3	601568	actin-binding cortex component	CPSQ3	617008	AR
SPTAN1 (αII)	182810	spectrin; actin-binding cortex component	DEE5	613477	AD
SPTBN1 (βII)	619475		DDISBA	619475	AD
SPTBN2 (βIII)	604985		SCA5	600224	AD
			SCA14	615386	AR
SPTBN4 (βIV)	606214		NEDHND	617519	AR
ANK3	600465	membrane-cytoskeleton linker	MRT37	615493	AR
ACTB	102630	actin	BRWS1	243310	AD
			DJO	607371	AD
ACTG1	102560	actin nucleation	BRWS2	614583	AD
			DFNA20	604717	AD
ARHGEF2	607560	Rho-GTP activator	NEDMHM	617523	AR
INF2	610982	actin-regulating formin (involved in mitochondrial fission)	CMTDIE	614455	AD
PFN1	176610	promoter of actin polymerisation (potential MT regulator)	ALS18	614808	-
DIAPH3	614567	formin, actin nucleation & polymerisation	AUNA1	609129	AD
GSN	137350	actin monomer-binding	amyloidosis	105120	AD
**C) Mitochondria & peroxisomes**					
DNM1L	603850	mitochondrial/proxisomal fission	OPA5	610708	AD
			EMPF1	614388	AD/R
MFF	614785	mitochondrial/peroxisomal fission	(PN) EMPF2	617086	AR
OPA1	605290	mitochondrial fusion	OPA1	165500	AD
MFN2	608507	mitochondrial fusion	CMT2A2A	609260	AD

			CMT2A2B	617087	AR
			HMSN6A	601152	AD
MPV17	137960	Multifunctional mitochondr. Inner membr. Prot.	CMT2EE	618400	AR
TIMM8A	300356	mitochondrial intermembrane space protein	MTS	304700	XLR
GDAP1	606598	mitochondrial dynamics	CMT4A	214400	AR
			CMTRIA	608340	AR
			CMTVCP	607706	AR
			CMT2K	607831	AD/R
SLC25A46	610826	lipid carrier at MCS with ER	HMSN6B	616505	AR
			PCH1E	619303	AR
POLG2	604983	mitochondrial DNA polymerase	MTDPS16B	619425	AR
CLPB	616254	mitochondrial AAA+ protease	MGCA7B	616254	AR
			MGCA7A	619835	AD
CLPP	601119		PRLTS3	614129	AR
SURF1	185620	assembly factor of mitochondrial complex IV	CMT4K	616684	AR
			MC4DN1 (ataxia)	220110	AR
DHTKD1	614984	mitochondrial dehydrogenase complex component	CMT2Q	615025	AD
PDK3	300906	mitochondrial pyruvate dehydrogenase kinase	CMTX6	300905	XLD
AIFM1	300169	mitochondrial FAD-dependent oxidoreductase, oxidative phosphorylation (also pro-apoptotic factor)	CMTX4	310490	XR
			COXPD6 (PN)	300816	XR
			DFNX5 (PN)	300614	XR
			SEMDHL	300232	XR
HPDL	618994	(mitochondrial?) potential dioxygenase	SPG83	619027	AR
			NEDSWMA	619026	AR
IBA57	615316	mitochondrial iron-sulfur cluster assembly pathway	SPG74	616451	AR
			MMDS3	615330	AR
HSPD1	118190	(mitochondrial) heatshock protein	SPG13	605280	AD
			HLD4	612233	AR
ALDH18A1	138250	mitochondrial aldehyde dehydrogenase	SPG9B	616586	AR
			SPG9A	601162	AD
MTRFR / C12ORF65	613541	mitochondrial translation release factor	SPG55	615035	AR
			COXPD7	613559	AR
DDHD1	614603	phospholipase A1 in mitochondria and ER	SPG28	609340	AR
SPG7 / PGN	602783	mitochondrial metalloprotease	SPG7	607259	AD/R
CHCHD2	616244	mitochondrial intermembrane protein (shuttling to nucleus)	PARK22	616710	AD
CHCHD10	615903		FTDALS2	615911	AD
			SMAJ	615048	AD
FXN	606829	mitochondrial iron chaperone involved in iron-sulfur biogenesis	FRDA (ataxia)	229300	AR
MICU1	605084	regulator of mitochondrial calcium uniporter (MCU)	MPXPS	615673	AR

PINK1	608309	Serine/threonine-protein kinase	PARK6	605909	AR
PARK2 / Parkin	602544	component of a multiprotein E3 ubiquitin ligase	PARK2	600116	AR
PDHA1	300502	pyruvate dehydrogenase	PDHAD	312170	XD
PDHB	179060	pyruvate dehydrogenase	PDHDB	614111	AR
PDHX	608769	PDH complex component	PDHXD	245349	AR
MPC1	614738	pyruvate carrier	MPYCD	614741	AR
GLUD2	300144	mitochondrial Glu dehydrogenase	PD	168600	AD
PISD	612770	formation of phosphatidylethanolamine	LIBF	618889	AR
MICOS13 / QIL	616658	MICOS complex, cristae regulation	COXPD37	618329	AR
OGDH	613022	oxoglutarate/α-ketoglutarate dehydrogenase (citrate cycle)	OGDHD	203740	AR
SUCLA2	603921	succinate-CoA ligase (citrate cycle)	MTDPS5	612073	AR
FH / Fumarase	136850	fumarate hydratase (citrate cycle)	FMRC	606812	AR
MDH1	154200	malate dehydrogenase (citrate cycle)	DEE88	618959	AR
MDH2	154100		DEE51	617339	AR
NDUFS4	602694	NADH:ubiquinone oxidoreductase, Complex I	MC1DN1	252010	AR
SDHA	600857	succinate dehydrogenase complex subunits (citrate cycle and complex II of ETC)	MC2DN1	252011	AR
			NDAXOA	619259	AD
SDHB	185470		MC2DN4	619224	AR
SDHD	602690		MC2DN3 (ataxia)	619167	AR
UQCRQ	612080	subunits of complex III of the ETC	MC3DN4	615159	AR
UQCRC1	191328		PKNPY	619279	AD
COX4I1	123864	subunits of complex IV of the ETC	MC4DN16	619060	AR
COX6A1	602072		CMTRID	616039	AR
COX8A	123870		MC4DN15	619059	AR
SURF1	185620	assembly factor of complex IV	MC4DN1	220110	AR
			CMT4K	616684	AR
ATP5F1A	164360	subunits of complex V of ETC (F_OF_1-ATP synthase subunit)	MC5DN4	615228	AR
ATP5F1E	606153		MC5DN3 (PN)	614053	AR
ATP5MC3	602736		DYTSPG	619681	AD
VPS13C	608879	mitophagy regulation	PARK23	616840	AR
VPS13D	608877	mitochondrial fission / mitophagy	SCAR4	607317	AR
WFS1	606201	TM regulator of Ca^{2+} homeostasis (also in ER)	Wolfram syndr. 1	222300	AR
CISD2	611507	TM protein at mitochondrial-ER contact sites	Wolfram syndr. 2	604928	AR
HTRA2	606441	serine peptidase, intermembrane	MGCA8	617248	AR
DTYMK	188345	deoxythymidine diphosphate synthesis	CONPM	619847	AR
MARS2	609728		SPAX3	611390	AR

		mitochondrial Met-tRNA synthetase 2	COXPD25	616430	AR
IARS2	612801	mitochondrial Ile-tRNA synthetase 2	CAGSSS (PN)	616007	AR
FARS2	611592	mitochondrial Phe-tRNA synthetase	SPG77	617046	AR
			COSPD14	614946	AR
EARS2	612799	mitochondrial Glu-tRNA synthetase	COXPD12	614924	AR
VARS2	612802	mitochondrial Val-tRNA synthetase	COXPD20	615917	AR
POLG	174763	mitochondrial DNA polymerase subunit	MTDPS4A	203700	AR
			SANDO (PN)	607459	AR
			PEOA1 (PN)	157640	AD
			PEOB1 (PN)	258450	AR
AARS2	612035	mitochondrial Ala-tRNA synthetase	LKENP	615889	AR
DARS2	610956	mitochondrial Asp-tRNA synthetase	LBSL	611105	AR
RARS2	611524	mitochondrial Arg-tRNA synthetase	PCH6	611523	AR
HSD17B4	601860	peroxisomal beta-oxidation	PRLTS1	233400	AR
ACOX1	609751	peroxisomal acyl-CoA oxidase	MITCH	618960	AD
FAR1	616107	peroxisomal fatty acyl-CoA reductase 1	CSPSD	619338	AD
ACBD5	616618	peroxosimal, processes long-chain acyl-CoA	RDLKD	618863	AR
SCP2	184755	peroxisomal thiolase	LKDMN	613724	AR
PEX1	602136	peroxisomal AAA ATPase complex component	CHR (Zellweger)	214100	AR
			PBD1B	601539	AR
GNPAT/DHAPAT	602744	peroxisomal lipid metabolism enzyme	RCDP2	602744	AR
AGPS	603051		RCDP3	600121	AR
AMACR	604489		AMACRD	614307	AR
ABCD1	300100	acyl-CoA transporter	ALD	300371	XR
ACBD5	616618		RDLKD	618863	AR
**D) Endosome & autophagy**					
RAB7A	602298	endocytic trafficking	CMT2B	600882	AD
GDI1	300104	Rab GEF	XLID41	300104	XD
SNX14	616105	endocytic trafficking, lipid droplet formation	SCAR20	616354	AR
SBF2	607697	GEF for RAB21 and possibly RAB28	CMT4B2	604563	AR
ALS2	606352	GEF of Rab5	ALSJ	205100	AR
			PLSJ	606353	AR
			IAHSP	607225	AR
SPG11 / spatacsin	610844	spatacsin, axonal growth, endocytic trafficking, autophagy	CMT2X	616668	AR
			ALS5	602099	AR
			SPG11	604360	AR
DNM2	602378	GTPase mediating scission during endocytosis and mitochondrial fission	CMTDIB / CMT2M	606482	AD
			LCCS5	615368	AR
AP4B1	607245	AP-4 complex subunit, endocytic trafficking	SPG47	614066	AR

AP4M1	602296	AP-4 complex component, endocytic trafficking	SPG50	612936	AR
VPS37A	609927	ESCRT-I component, endocyt. Trafficking	SPG53	614898	AR
UBAP1	609787	ESCRT-I complex	SPG80	618418	AD
SPART	607111	MT-associated, regulates endocytic traffic/mitochondria	SPG20 / Troyer syndrome	275900	AR
AP4S1	607243	AP-4 complex component, endocytic trafficking	SPG52	614067	AR
ZFYVE26 / spastizin	612012	Phosphatidylinositol 3-phosphate-binding protein, lysosomal, cytoskeletal	SPG15	270700	AR
TECPR2	615000	potential positive regulator of autophagy	HSAN9 SPG49	615031	AR
NIPA1	608145	magnesium transporter, PM, endosome	SPG6	600363	AD
AP4E1	607244	adaptor protein complex-4, endocytic trafficking	SPG51	613744	AR
ARL6IP1	607669	endocytic trafficking	SPG61	615685	AR
CHMP1A	164010	ESCRT-III complex component	PCH8	614961	AR
CHMP2B	609512		FTDALS7/FTD3	600795	AD
OPTN	602432	autophagy receptor	ALS12	613435	AD/R
			POAG	137760	AD
TBK1	604834	kinase of OPTN	FTDALS4	616439	AD
SQSTM1 / p62 / sequestosome	601530	autophagy adaptor	FTDALS3	616437	AD
			NADGP (ataxia)	617145	AR
UBQLN2	300264		ALS15	300857	XD
HTT	613004	regulator of endomembrane dynamics	HD	143100	AD
			LOMARS	617435	AR
WASHC4	615748	actin nucleation on endosomes	MRT43	615817	AR
WASHC5 / Strumpellin	610657	WASH core complex component, actin regulation	SPG8	603563	AD
			RTSC1	220210	AR
TMEM106B	613413	lysosomal TM protein	HLD16	617964 [Ref.4]	AD
RAB3GAP1	602536	Rab3-mediated exocytosis	MARTS2	619420	AR
			WARBM1	600118	AR
RAB3GAP2	609275	Rab3-mediated exocytosis	MARTS1	212720	AR
			WARBM2	614225	AR
TBC1D20	611663	Rab1/2 GAP, vesicular transport	WARBM4	615663	AR
TBC1D2B	619152	GAP for Rab22	NEDSGO	619323	AR
VPS4A	609982	ESCRT-III regulator	CIMDAG	619273	AD
VPS16	608550	early/late endosome conversion	DYT30	619291	AD
VPS41	605485	endo-/auto-/phagosomal trafficking	SCAR29	619389	AR
VPS35	601501	retromer complex component	PARK17	614203	AD
MCOLN1	605248	endo/lysosomal non-selective ion channel	ML IV	252650	AR
ATG5	604261	autophagosome formation	SCAR25	617584	AR

ATG7	608760	autophagosome transport	SCAR31	619422	AR
WDR81	614218	endo-/lysosomal trafficking	CAMRQ2 (ataxia)	610185	AR
			HYC3	617967	AR
LRRK2	609007	lysosomal/ER/mitochondrial kinase & scaffold	PARK8	607060	AD
PPT1	600722	lysosomal enzyme	CLN1	256730	AR
TPP1	607998		CLN2	204500	AR
CLN3	607042	endo-/lysosomal TM protein	CLN3	204200	AR
CLN5	608102	endo-/lysosomal TM protein	CLN5	256731	AR
CLN6	606725	endosomal/ER TM protein	CLN6A	601780	AR
			CLN6B	204300	AR
MFSD8	611124	lysosomal TM transporter	CLN7	610951	AR
CTSD	116840	cathepsins; lysosomal papain family protease	CLN10	610127	AR
CTSF	603539		CLN13	615362	AR
BIN1 / AMPH2	601248	endosomal membrane shaping	CNM2	255200	AR
CLCN3	600580	endo-/lysosomal/PM Cl-channel/transporter	NEDHYBA	619512	AD
CLCN6	602726	endosomal Cl^-/H^+ antiporter	CONRIBA	619173	AD
WIPI2	609225	autophagy regulators	IDDSSA	618453	AR
WDR45B / WIPI3	609226		NEDSBAS	617977	AR
WDR45 / WIPI4	300526		NBIA5	300894	XD
NPC1	607623	endo-lysosomal cholesterol transporter	NPC C1/D	257220	AR
NPC2	601015		NPC C2	607625	AR
APOE	107741	lipid transporter	AD2	104310	AD
GBA	606463	lysosomal enzyme, glycolipid catabolism	DLB	127750	AD
			PD	168600	AD
			GD2	230900	AR
			GD3	231000	AR
			GD3C	231005	AR
GRN / progranulin	138945	secreted protein, lysosomal regulator	FTLDU	607485	AD
			CLN11	614706	AR
GALC	606890	lysosomal enzyme	KRB	245200	AR
MCOLN1 / TRPML1	605248	lysosomal cation channel	ML4	252650	AR
ATP13A2	610513	lysosomal cation transporter	SPG78	617225	AR
			KRS / PARK9	606693	AR
FIG4	609390	endosomal PtdIns(3,5)P2 5-phosphatase	ALS11	612577	AD
			BTOP	612691	AR
			CMT4J	611228	AR
NAGLU	609701	lysosomal glucosaminidase, degrades heparan sulfate	CMT2V	616491	AD
ATP6V1A	607027	V-ATPase subunit (V_1)	DEE93	618012	AD
ATP6V1B2	606939		ZLS2	616455	AD
ATP6V0A1	192130	V-ATPase subunit (V_0)	DEE104	619970	AD
			NEDEBA	619971	AR
ATP6AP2	300556		XPDS	300911	XR
			MRXSH	300423	XR
**E) Golgi & ER**					
SELENOI	607915	ER/Golgi-based, phosphatidylethanolamine	SPG81	618768	AR

		biosynthesis important for vesicles			
REEP1	609139	ER morphogenesis	SPG31	610250	AD
			HMN5B	614751	AD
REEP2	609347	tubular ER morphogenesis, ER-MT interaction	SPG72	615625	AD/R
REEP6	609346	ER homeostasis	RP77	617304	AR
C19ORF12	614297	ER morphogenesis	SPG43	615043	AR
			NBIA4	614298	AD/R
TFG	602498	ER to Golgi trafficking	SPG57	615658	AR
			HMSNO	604484	AD
SLC33A1	603690	acetyl-CoA Transporter (PM, ER, Golgi)	SPG42	612539	AD
			CCHLND	614482	AR
CYP2U1	610670	monooxygenase, arachidonic acid metabolism (mitochondria, ER)	SPG56	615030	AR
ERLIN2	611605	ER-associated degradation of IP3Rs	SPG18	611225	AR
DDHD2	615003	phospholipase enzyme; ER/Golgi	SPG54	615033	AR
CYP7B1	603711	ER-associated cytochrome P450 monooxygenases	SPG5A	270800	AR
GBA2	609471	Non-lysosomal glucosylceramidase; ER, Golgi, cytosol	SPG46	614409	AR
ZFYVE27 / Protrudin	610243	regulator of RAB11-dependent vesicular trafficking and ER morphogenesis	SPG33	610244	AD
ERLIN1	611604	ER-associated degradation of IP3Rs	SPG62	615681	AR
CLN8	600143	ER TM lipid regulator	CLN8	600143	AR
BSCL2 / Seipin	606158	lipid droplet formation	PELD	615924	AR
			HMN5C	619112	AD
			SPG17	270685	AD
B4GALNT1	601873	Golgi-based GalNAc-T, biosynthesis of glycosphingolipids	SPG26	609195	AR
ATL1	606439	GTPase, ER/Golgi biogenesis, lipid droplet regulation*	SPG3A	182600	AD
			HSN1D	613708	AD
ATL3	609369	tubular ER morphogenesis*	HSN1F	615632	AD
FA2H	611026	ER-based, sphingolipid biogenesis, (myelin production?)	SPG35	612319	AR
PCYT2	602679	phospholipid synthesis, ER-based	SPG82	618770	AR
PNPLA6	603197	intra-ER phospholipase	SPG39	612020	AR
			LNMS	245800	AR
			OMCS	275400	AR
			BNHS (ataxia)	215470	AR
RTN2	603183	ER morphogenesis	SPG12	604805	AD

CPT1C	608846	ER/mitochondrial acetyl-transferase regulating fatty acids	SPG73	616282	AD
SIGMAR1	601978	lipid transport from the ER	ALS16	614373	AR
			DSMA2 (PN)	605726	AR
VCP	601023	regulator of Golgi and ER dynamics / activator of autophagy [Ref.6]	FTDALS6	613954	AD
			CMT2Y	616687	AD
			IBMPFD1	167320	AD
VAPBC	605704	sphingolipid metabolism, ER unfolded protein response	ALS8	608627	AD
			SMAFK	182980	AD
ARL6IP1	607669	TM, tubular ER morphogenesis*	SPG61	615685	AR
ATXN2	601517	membrane-associated, tubular ER morphogenesis*	SCA2 ALS13	183090	AD
			PD	168600	AD
ITPR1 / IP3R1	147265	Inositol 1,4,5-triphosphate-gated calcium channel	GLSP (ataxia)	206700	AD/R
			SCA15	606658	AD
			SCA29	117360	AD
JPH1	605266	ER-PM MCS component	CMT2K	607831	AD/R
JPH3	605268	ER-PM MCS component	HDL2	606438	AD
PDZD8	614235	ER-mito MCS component	IDDADF	620021	AR
ATAD3A	612316	ER-mito MCS component	HAYOS	617183	AD/R
			PHRINL	618810	AR
LNPK	610236	ER-shaping membrane protein	NEDEHCC	618090	AR
NBAS / NAG	608025	Golgi-to-ER transport	SOPH	614800	AR
Rab18	602207	formation of ER-LD contacts	WARBM3	614222	AR
VPS13A	605978	ER-mitochondria contact sites	CHAC	200150	AR
LSS	600909	cholesterol synthesis	APMR4	618840	AR
**F) Cytoplasm, nucleus & plasma membrane (only randomly selected)**					
SOD1	147450	superoxide to H_2O_2 catalysis	ALS1	105400	AD/R
			STAHP	618598	AR
HK1	142600	glycolysis-related enzyme	HMSNR CMT4G	605285	AR
			NEDVIBA	618547	AD
GPT2 / ALT2	138210	L-alanin to pyruvate catalysis	NEDSPM	616281	AR
DJ1	602533	oxidative stress sensor	PARK7	606324	AR
GARS1	600287	glycyl-tRNA synthetase	HMN5A	600794	AD
			CMT2D	601472	AD
			SMAJI	619042	AD
PGK1	311800	phosphoglycerate kinase	PGK1 deficiency	300653	XR
SARM1	607732	NAD^+ hydrolase	ALS	[Ref.9]	AD?
NMNAT1	608700	NMN adenylyltransferase	LCA9	608553	AR
			SHILCA	619260	AR
NMNAT2	608701		polyneuropathy	[Ref.10]	?
GAN	605379	E3 ubiquitin-protein ligase complex adaptor	GAN	256850	AR
HSPB1	602195	heat shock protein, chaperone	CMT2F	606595	AD
GPX4	138322	glutathione peroxidase	SMDS	250220	AR
ATXN1	601556	cytoplasmic, RNA-binding	SCA1	164400	AD

ATXN8OS	603680	non-coding RNA gene	SCA8	608768	AD
			PD	168600	AD
ATXN3	607047	deubiquitinating enzyme	MJD (ataxia)	109150	AD
ATXN7	607640	STAGA transcription coactivator-HAT complex component	SCA7	164500	AD
INPP5K	607875	Inositol Polyphosphate-5-Phosphatase K	MDCCAID	617404	AR
GSS	601002	glutathione synthetase	GSSD	266130	AR
GCLC	606857	glutathione synthesis	GGSD	230450	AR
ATP2B3	300014	ATP-driven calcium pump	SCAX1	302500	XR
ABCA1	600046	lipid export (PM & endosomes)	(PN) TGD	600046	AR
IREB2	147582	cytosolic regulator of ferritin mRNA	NDCAMA	618451	AR
ADH1C	103730	alcohol dehydrogenase	PD	168600	AD/Mu
TBP	600075	transcription factor	SCA17	607136	AD
TARDBP / TDP43	605078	RNA-binding protein	ALS10	612069	AD
FUS	137070	DNA/RNA-binding protein	ALS6	608030	-
RELN	600514	Extracellular matrix serine protease	LIS2	257320	AR
SLC2A1/GLUT1	138140	PM glucose transporter	DYT9	601042	AD
			GLUT1DS1	606777	AD/R
SLC1A4	600229	PM amino acid transporter	SPATCCM	616657	AR
SLC16A2/MCT8	300095	PM monocarboxylate transporter	AHDS (SPG)	300523	X?
TTC5	619014	tubulin-specific ribosome-associating factor	NEDCAFD	619244	AR
DNAJC5/CSP	611203	synaptic chaperone	CLN4	162350	AD
PSMC1	602706	proteasome subunit	NEDGTH	620071	AR
PSMC3	186852		DCIDP	619354	AR
PSMD12	604450		STISS	617516	AD
PLEKHG5	611101	NFκB signalling	CMTRIC	615376	AR
			DSMA4	611067	AR
YARS1	603623	Aminoacyl-tRNA synthetase	CMTDIC	608323	AD
ATP1A1	182310	Na/K-ATPase ion pump	CMT2DD	618036	AD
			HOMGSMR2	618314	AD
MME	120520	membrane metalloendopeptidase	CMT2T	617017	AD/R
			SCA43	617018	AD
GNB4	610863	heterotrimeric G protein subunit	CMTDIF	615185	AD
TRIM2	614141	E3 ubiquitin ligase	CMT2R	615490	AR
HARS1	142810	catalyzes the covalent ligation of histidine to its cognate tRNA	CMT2W	616625	AD
			USH3B	614504	AR
LRSAM1	610933	RING finger ubiquitin ligase regulating cell adhesion molecules	CMT2P	614436	AD/R
IGHMBP2	600502	helicase, transcription regulation	CMT2S	616155	AR
			DSMA1 HMN6	604320	AR
MTMR2	603557	phosphoinositide lipid phosphatase	CMT4B1	601382	AR

FGD4	611104	frabin, CDC42 GEF, actin-binding	CMT4H	609311	AR
MARS1	156560	methionyl-tRNA synthetase	CMT2U	616280	AD
TRPV4	605427	Ca^{2+} channel responding to physical, chemical, hormonal stimuli	HMS N2C / CMT2C	606071	AD
			HMN8	600175	AD
			SPSMA	181405	AD
HSPB8	608014	heatshock protein	CMT2L	608673	AD
			HMN2A	158590	AD
FMR1	309550	RNA-binding protein	FXS	300624	XD
AARS1	601065	alanyl-tRNA synthetase	CMT2N	613287	AD
			DEE29	616339	AR
KARS1	601421	lysyl-tRNA synthetase	CMTRIB	613641	AR
PNKP	605610	phosphatase in DNA repair	CMT2B2	605589	AR
			AOA4	616267	AR
			MCSZ	613402	AR
MORC2	616661	DNA repair, gene regulation	CMT2Z	616688	AD
SBF1	603560	regulates MTMR2 phosphatase, potentially a Rab28 GEF	CMT4B3	615284	AR
PRPS1	311850	purine/pyrimidine biosynthesis	CMTX5	311070	XR
			ARTS	301835	XR
			DFNX1	304500	X?
			SUPERACTIVITY	300661	XR
AMPD2	102771	purine metabolism, converting AMP to IMP	SPG63	615686	AR
			PCH9	615809	AR
DSTYK	612666	PM tyrosine kinase, cell death regulation	SPG23	270750	AR
GJB1	304040	connexin, gap junction protein	CMTX1	302800	XD
GJC2	608803	gap junction protein	SPG44	613206	AR
			HLD2	608804	AR
PRNP	176640	GPI-anchored membrane protein	GSD	137440	AD
			CJD	123400	AD
			HDL1	603218	AD
			Spongiform encephalopathy	606688	AD
SYNJ1	604297	synaptic endocytosis	PARK20	615530	AR
PCLO	604918	presynaptic matrix protein	PCH3	608027	AR
KCNA2 / Kv1.2	176262	voltage-gated potassium channel subunit	DEE32	616366	AD
KCNA4 / Kv1.4	176266		MCIDDS	618284	AR
KCNC3 / Kv3.3	176264		SCA13	605259	AD
KCND3 / Kv4.3	605411		SCA19	607346	AD
CACNA1A / CaV2.1	601011	voltage-gated calcium channel subunit	SCA6	601011	AD
			DEE42	617106	AD
CACNA1G / Cav3.1	604065		SCA42	616795	AD
CACNA2D1	114204		DEE110	620149	AR
SCN1A / Nav1.1	182389	voltage-gated sodium channel subunit	DEE6B	619317	AD
SCN1B	600235		DEE52	617350	AR

SCN11A	604385		HSAN7	615548	AD
UCHL1	191342	cytosolic thiol protease	SPG79	615491	AR
			PARK9	613643	AD
AP5Z1	613653	DNA double-strand break repair, endosomal transport	SPG48	613647	AR
ENTPD1	601752	PM hydrolyse, regulates purinergic neurotransmission	SPG64	615683	AR
NT5C2	600417	hydrolase for purine nucleotides	SPG45	613162	AR
CAPN1	114220	Calpain, calcium-sensitive cysteine protease	SPG76	616907	AR
L1CAM	308840	cell adhesion molecule	SPG1	303350	XR
			PACC	304100	XR
ERBB4	600543	EGF receptor tyrosine kinase	ALS19	615515	AD
NEK1	604588	cytosolic kinase involved in DNA damage checkpoint control	ALS24	617892	AD
MATR3	164015	nuclear matrix protein, transcriptional/RNA regulation?	ALS21	606070	AD
C9orf72	614260	E3 ubiquitin-protein ligase complex	FTDALS1	105550	AD
SETX	608465	RNA metabolism, genomic integrity	ALS4	602433	AD
			SCAN2	606002	AR
HNRNPA1	164017	mRNA regulation	ALS20	615426	AD
ANG	105850	ribosomal and tRNA regulation	ALS9	611895	?
SPG11	610844	transmembrane protein phosphorylated upon DNA damage	ALS5	602099	AR
			CMT2X	616668	AR
			SPG11	604360	AR
MAOA	309850	oxidative deamination of amines (e.g. dopamine, serotonin)	BRNRS	300615	XR

Tab. S1 OMIM-listed genes linked to neurodegenerative or neurodevelopmental conditions which can be expected to cause developmental and/or degenerative axonal aberrations. Rows are grouped by cell biological contexts as indicated in grey bars. 1st column: HGNC-approved symbols of disease-linked genes, hyperlinked to genecards.org; 2nd column: OMIM reference number for each gene hyperlinked to omim.org; 3rd column: brief description of gene function (PM, plasma membrane; TM, transmembrane); 4th and 5th column: acronym of linked diseases and their references (hyperlinked OMIM reference number or published case study); colour code indicates disorder classification, as explained above the table; 6th column: form of inheritance: A, autosomal; D, dominant; R, recessive; XL, X-linked. References: 1 - Boyle et al., 2021; 2 - Nicita et al., 2020; 3 - Goizet et al., 2009; Crimella et al., 2012; 4 - Feng et al., 2022; 5 - Miyake et al., 2016; 6 - Wrobel et al., 2022; 7 - Edvardson et al., 2016; 8 - Bayrakli et al., 2015); 9 - Gilley et al., 2021;Bloom et al., 2022; 10 - Huppke et al., 2019;Lukacs et al., 2019). Further abbreviations: PN, peripheral neuropathy; SP, spastic paraplegia.

ACTR = ARP	actin-related protein
acyl-/acetyl-CoA	acyl-/acetyl-linked co-enzyme A
AD	Alzheimer's disease
ADPR	adenosine diphosphate ribose
ALS	amyotrophic lateral sclerosis
ANT = SLC25A4	ADP/ATP translocator of skeletal muscle = solute carrier family 25, member 4
ARL6IP	ADP-ribosylation factor-like GTPase 6-interacting protein
ARL	ADP-ribosylation factor-like GTPase
ATG	autophagy-related
ATP	adenosine triphosphate
ATF	activating transcription factor
ATP2A = SERCA	ATPase, Ca^{2+}-transporting, fast twitch = sarcoplasmic reticulum Ca^{2+}-ATPase
ATP2B = PMCA	ATPase, Ca^{2+}-transporting, plasma membrane = plasma membrane Ca^{2+}-ATPase
BIN = AMPH	bridging integrator = amphiphysin
BLOC	biogenesis of lysosome-related organelles complex
BORC	BLOC1-related complex
cADRP	cyclic adenosine diphosphate ribose
CHMP	charged multivesicular body protein
CKAP	cytoskeleton-associated protein
CLIMP	cytoskeleton-linking membrane protein
CMT	Charcot-Marie-Tooth disease
C9ORF72	chromosome 9 open reading frame 72
CYCS	cytochrome C, somatic
DENND	DENN/MADD domain-containing protein
Dhc	dynein heavy chain (*Drosophila*)
DNM1L = DRP1	dynamin-like = dynamin-related protein
EB/Eb	MT end-binding protein (≈ MAPRE); human/*Drosophila*
Efa6	Exchange factor for Arf 6
EIF2AK3 = PERK	eukaryotic translation initiation factor 2α kinase 3 = pancreatic EIF2α kinase
ER	endoplasmic reticulum
ERN	endoplasmic reticulum-to-nucleus signaling
ESCRT	endosomal sorting complex required for transport
FAD	flavin adenine dinucleotide
FTDALS4	frontotemporal dementia and/or amyotrophic lateral sclerosis
GALC	galactosylceramidase
GAN	giant axonal neuropathy
GBA	glucosidase, beta, acid
GSSG	reduced glutathione
GTP	guanosine triphosphate
HGNC	HUGO Gene Nomenclature Committee
HSP/SPG	hereditary spastic paraplegia
IRE	inositol-requiring enzyme
JIP = MAPK8IP	JNK-interacting protein = mitogen-activated protein kinase 8-interacting protein
Khc	Kinesin heavy chain (*Drosophila*)

KIF	kinesin family member
KLC/Klc	kinesin light chain (human/*Drosophila*)
LAMP	lysosome-associated membrane protein
LC3	see MAP1LC3
MACF1	microtubule-actin cross-linking factor
MAP1LC3	microtubule-associated protein 1, light chain 3 (=LC3/Atg8)
MAP6	microtubule associated protein 6
MAPRE	microtubule-associated protein, RP/EB family
MAPT/tau	microtubule-associated protein tau (human/*Drosophila*)
MCU	mitochondrial calcium uniporter complex
MFN	mitofusin
mPTP	mitochondrial permeability transition pore
MPTP	methyl-phenyl-tetrahydropyridine; prodrug to the neurotoxin MPP^+
MT	microtubule
mtDNA	mitochondrial DNA
MUCOLN = TRPML	mucolipidin / transient receptor potential cation channel, mucolipin subfamily, member
MVB	multivesicular body
NAD	nicotinamide adenine dinucleotide
NMNAT	nicotinamide nucleotide adenylyltransferase
NPC1	Nieman-Pick C intracellular cholesterol transporter
OPA	optic atrophy
OPTN	optineurin
OXPHOS	oxidative phosphorylation
p62	ubiquitin-binding protein 62
PARK	parkin
PICALM	phosphatidylinositol-binding clathrin assembly protein
POLG	DNA Polymerase Gamma
PSMF1 = PI31	proteasome inhibitor subunit 1 = proteasome inhibitor, 31-kD
RAB	RAB-associated protein
REEP	receptor expression-enhancing protein
RHOT/Miro	RAS homologue T / mitochondrial Rho (*Drosophila*)
RILP	RAB-interacting lysosomal protein
ROS	reactive oxygen species
RRBP1 = P180	ribosome binding protein 1
RTN	reticulon
RYR	ryanodine receptor
SARM	sterile alpha and TIR motifs-containing protein
SKIIP	SKI-interacting protein
SNAPIN	SNAP-associated protein
SOD	superoxide dismutase
SPOAN	spastic paraplegia, optic atrophy, and neuropathy
SLC8A = NCX	solute carrier family 8 = sodium calcium exchanger
STIM	stromal interaction molecule
STRIPAK	striatin-interacting phosphatase and kinase
TBK	TANK-binding kinase
TRAK/Milton	trafficking protein, kinesin-binding / *Drosophila* homologue
TRPM	transient receptor potential cation channel, subfamily M

VDAC	voltage-dependent anion channel
VPS	vacuolar protein sorting
WDR	WD repeat-containing protein

Tab. S2 Abbreviations used in the main text. All human/*Drosophila* gene symbols follow HGNC/FlyBase-approved nomenclature.

**Figure 2**		
**ACLY**	ATP CITRATE LYASE	108728
**(c)ADPR**	cyclic/adenosine diphosphate ribose	---
SLC25A4/5 / **ANT**	SOLUTE CARRIER FAMILY 25	103220; 300150
SLC1A4 / **ASCT1**	SOLUTE CARRIER FAMILY 1 MEMBER 4/NEUTRAL AMINO ACID TRANSPORTER	600229*
**F_OF_1** ATP synthase components linked to neural disorders	**ATP5F1A**: ATP SYNTHASE F1, SUBUNIT ALPHA	164360*
	**ATP5F1E**: ATP SYNTHASE F1, SUBUNIT EPSILON	606153*
**1,3 BPG**	1,3-biphosphoglycerate	---
**citrate cycle** components linked to neural disorders	**OGDH:** OXOGLUTARATE/Ketoglutarate DEHYDROGENASE	613022*
	**SUCLA2:** SUCCINATE-CoA LIGASE	603921*
**CYCS**	CYTOCHROME C	123970
**ETC**	electron transfer chain	---
**DCA**	dichloroacetate; clinical drug that upregulates PDH activity; causing peripheral neuropathy as side effect;)(Tataranni and Piccoli, 2019)	---
**FAD**	flavin adenine dinucleotide	---
**FBP**	fructose 1,6 biphosphate	---
**FH** / Fumarase	FUMARATE HYDRATASE	136850
**GAP**	glyceraldehyde-3-phosphate	---
**GAPDH**	GLYCERALDEHYDE-3-PHOSPHATE DEHYDROGENASE	138400
SLC2A / **GLUT**	SOLUTE CARRIER FAMILY 2/GLUCOSE TRANSPORTER	138140*; 138160; 138170; 138190
**GPT**	GLUTAMATE PYRUVATE TRANSAMINASE	138200; 138210*
**HK**	HEXOKINASE	142600*; 601125; 142570
**MPC**	MITOCHONDRIAL PYRUVATE CARRIER	614738*; 614737
**LDH**	LACTATE DEHYDROGENASE	150000; 150100; 150150
SLC16A / **MCT**	SOLUTE CARRIER FAMILY 16/MONOCARBOXYLATE TRANSPORTER	600682; 300095*; 603877; 603878; 603879; 603880; 603654; 610409; 614242; 607550
**MDH**	MALATE DEHYDROGENASE	154200; 154100
**NA**	nicotinamide	---
**NAD**	nicotinamide adenine dinucleotide	---
**NMN**	nicotinamide mononucleotide	---
**NMNAT**	NICOTINAMIDE NUCLEOTIDE ADENYLYLTRANSFERASE	608700*; 608701; 608702
**OXPHOS**	oxidative phosphorylation	---
**PDH**	PYRUVATE DEHYDROGENASE	300502*; 179060*; 608769*
**PFK**	PHOSPHOFRUCTOKINASE	---
**3-PGA**	3-phosphoglycerate	---
**PGK**	PHOSPHOGLYCERATE KINASE	311800*; 172270
**PK**	PYRUVATE KINASE	---
**Q**	quinone	---
**SARM1**	STERILE ALPHA AND TIR MOTIFS-CONTAINING PROTEIN	607732
**SDH** complex	SUCCINATE DEHYDROGENASE	600857; 185470; 602690

**Figure 3**		
**ANT** / SLC25	ADP/ATP TRANSLOCATOR/SOLUTE CARRIER FAMILY 25	103220; 300150
**ATP2A/SERCA**	Ca^{2+}-TRANSPORTING FAST-TWITCH ATPase	108730; 108740; 601929
**ATP2B** / PMCA	Ca^{2+}-TRANSPORTING ATPase	108731, 108733, 300014*
**BAK**	BCL2 ANTAGONIST KILLER	600516
**BAX**	BCL2-ASSOCIATED X PROTEIN	600040
**C2CD2** / TMEM24	C2 CALCIUM-DEPENDENT DOMAIN-CONTAINING PROTEIN	617581; 617582
**CC**	unspecified Ca^{2+} channel	---
**CYCS**	CYTOCHROME C, SOMATIC	123970
**DJ1**	ONCOGENE DJ1	602533*
**ESYT**	EXTENDED SYNAPTOTAGMIN-LIKE PROTEIN	616670; 616691; 616692
**F_OF_1** ATP synthase components linked to neural disorders	**ATP5F1A**: ATP SYNTHASE F1, SUBUNIT ALPHA	164360*
	**ATP5F1E**: ATP SYNTHASE F1, SUBUNIT EPSILON	606153*
**ITPR** / IP3R	INOSITOL 1,4,5-TRIPHOSPHATE RECEPTOR	147265*; 600144; 147267
**HSPA9** / GRP75	HEAT-SHOCK 70-KD PROTEIN 9/GLUCOSE-REGULATED PROTEIN	600548
**JPH**	JUNCTOPHILIN	605266*; 605267; 605268*
**MCU** complex	MITOCHONDRIAL CALCIUM UNIPORTER	614197; 616952; 605084*; 610632
**MICOS13** / QIL1	13kD subunit of MITOCHONDRIAL CONTACT SITE AND CRISTAE ORGANIZING SYSTEM (important for cisternae formation)	616658*;
**mPTP**	mitochondrial permeability transition pore	---
**MOMP**	mitochondrial outer membrane pore	---
**ORAI**	ORAI CALCIUM RELEASE-ACTIVATED CALCIUM MODULATOR	610929; 610277; 610930
**OSBPL** / ORP	OXYSTEROL-BINDING PROTEIN-LIKE PROTEIN	606733; 606736
**PC**	phosphatidylcholine	---
**PE**	phosphatidylethanolamine	---
**PISD** / PSD	PHOSPHATIDYLSERINE DECARBOXYLASE	612770*
**PL**	phospholipids	---
**PPID** / CYBD	PEPTIDYL-PROLYL ISOMERASE D / Cyclophilin D	601753
**PS**	phosphatidylserine	---
**PTDSS**	PHOSPHATIDYLSERINE SYNTHASE	612792; 612793
**PTPIP51** / FAM82A2	PROTEIN TYROSINE PHOSPHATASE-INTERACTING PROTEIN 51	611873
**RYR**	RYANODINE RECEPTOR	180901; 180902; 180903
**SAMM50** / SAM50	SAMM50 SORTING AND ASSEMBLY MACHINERY COMPONENT	612058
**SEC22**	SEC22 HOMOLOG	604028; 604029; 612442
**SOCE**	store-operated Ca^{2+} entry: STIM senses ER Ca^{2+} depletion and activates ORAI	---
**SLC8A** / NCX	SOLUTE CARRIER FAMILY 8	182305; 601901; 607991
**STIM**	STROMAL INTERACTION MOLECULE	605921; 610841
**STX1**	SYNTAXIN 1	186590; 186591
**TRPM2**	TRANSIENT RECEPTOR POTENTIAL CATION CHANNEL	603749

**VAPBC**	VAMP-ASSOCIATED PROTEIN C	605704*
**VDAC**	VOLTAGE-DEPENDENT ANION CHANNEL	604492; 193245; 610029; 610030
**VPS13**	VACUOLAR PROTEIN SORTING 13 HOMOLOG	605978*; 607817; 608879*; 608877*
**Figure 4**		
**ACOX**	ALDEHYDE OXIDASE 1, β-oxidisation	602841*; 601641; 603402
**CAT**	CATALASE	115500
**DEM**	diethyl maleate	---
**DUOX**	DUAL OXIDASE / MATURATION FACTOR	606758; 606759; 612771; 612772
**ETC**	electron transfer chain	---
**GCLC**	GLUTAMATE-CYSTEINE LIGASE	606857*
**GPX**	GLUTATHIONE PEROXIDASE	138319; 138321; 138322*; 603435; 607913; 615784; 617172
**GSH**	glutathione	---
**GSR**	GLUTATHIONE REDUCTASE	138300
**GSS**	GLUTATHIONE SYNTHETASE	601002*
**MPTP**	1-methyl-4-phenyl-1,2,3,6-tetrahydropyridine	---
**NAD**	nicotinamide adenine dinucleotide	---
**NOX** complex	NADPH OXIDASE / ACTIVATOR / ORGANIZER	300225; 300481; 611255; 607105; 605261; 606572; 608515; 611256; 608512
**PRDX**	PEROXIREDOXIN	176763; 600538; 604769; 300927
**SOD1**	CuZn-dependent SUPEROXIDE DISMUTASE	147450*
**SOD2**	Mn-dependent SUPEROXIDE DISMUTASE	147460
**SOD3**	CuZn-dependent SUPEROXIDE DISMUTASE	185490
**TXN**	THIOREDOXIN	187700
**TXNRD2**	THIOREDOXIN REDUCTASE 2	601112; 606448; 606235

Tab. S3 Abbreviations of gene names shown in the figures. Abbreviations for each figure are listed in alphabetic order. **1st column**: HGNC-approved symbol; **2nd column**: full gene names; **3rd column**: hyperlinked OMIM reference numbers for all paralogues; asterisks indicate disease-linked genes listed in Tab.S1.

Supplementary references

Bayrakli, F., Poyrazoglu, H.G., Yuksel, S., Yakicier, C., Erguner, B., Sagiroglu, M.S., Yuceturk, B., Ozer, B., Doganay, S., Tanrikulu, B., Seker, A., Akbulut, F., Ozen, A., Per, H., Kumandas, S., Altuner Torun, Y., Bayri, Y., Sakar, M., Dagcinar, A., and Ziyal, I. (2015). Hereditary spastic paraplegia with recessive trait caused by mutation in KLC4 gene. *J Hum Genet* 60**,** 763-768.

Bloom, A.J., Mao, X., Strickland, A., Sasaki, Y., Milbrandt, J., and Diantonio, A. (2022). Constitutively active SARM1 variants that induce neuropathy are enriched in ALS patients. *Mol. Neurodegener* 17**,** 1.

Boyle, L., Rao, L., Kaur, S., Fan, X., Mebane, C., Hamm, L., Thornton, A., Ahrendsen, J.T., Anderson, M.P., Christodoulou, J., Gennerich, A., Shen, Y., and Chung, W.K. (2021). Genotype and defects in microtubule-based motility correlate with clinical severity in KIF1A-associated neurological disorder. *Hum Genet Genom Adv* 2**,** 100026.

Crimella, C., Baschirotto, C., Arnoldi, A., Tonelli, A., Tenderini, E., Airoldi, G., Martinuzzi, A., Trabacca, A., Losito, L., Scarlato, M., Benedetti, S., Scarpini, E., Spinicci, G., Bresolin, N., and Bassi, M.T. (2012). Mutations in the motor and stalk domains of KIF5A in spastic paraplegia type 10 and in axonal Charcot-Marie-Tooth type 2. *Clin Genet* 82**,** 157-164.

Edvardson, S., Tian, G., Cullen, H., Vanyai, H., Ngo, L., Bhat, S., Aran, A., Daana, M., Da'amseh, N., Abu-Libdeh, B., Cowan, N.J., Heng, J.I.-T., and Elpeleg, O. (2016). Infantile neurodegenerative disorder associated with mutations in TBCD, an essential gene in the tubulin heterodimer assembly pathway. *Hum Mol Genet* 25**,** 4635-4648.

Feng, T., Luan, L., Katz, I.I., Ullah, M., Van Deerlin, V.M., Trojanowski, J.Q., Lee, E.B., and Hu, F. (2022). TMEM106B deficiency impairs cerebellar myelination and synaptic integrity with Purkinje cell loss. *Acta Neuropathologica Communications* 10**,** 33.

Gilley, J., Jackson, O., Pipis, M., Estiar, M.A., Al-Chalabi, A., Danzi, M.C., Van Eijk, K.R., Goutman, S.A., Harms, M.B., Houlden, H., Iacoangeli, A., Kaye, J., Lima, L., Queen Square, G., Ravits, J., Rouleau, G.A., Schüle, R., Xu, J., Züchner, S., Cooper-Knock, J., Gan-Or, Z., Reilly, M.M., and Coleman, M.P. (2021). Enrichment of SARM1 alleles encoding variants with constitutively hyperactive NADase in patients with ALS and other motor nerve disorders. *eLife* 10**,** e70905.

Goizet, C., Boukhris, A., Mundwiller, E., Tallaksen, C., Forlani, S., Toutain, A., Carriere, N., Paquis, V., Depienne, C., Durr, A., Stevanin, G., and Brice, A. (2009). Complicated forms of autosomal dominant hereditary spastic paraplegia are frequent in SPG10. *Human Mutation* 30**,** E376-E385.

Huppke, P., Wegener, E., Gilley, J., Angeletti, C., Kurth, I., Drenth, J.P.H., Stadelmann, C., Barrantes-Freer, A., Brück, W., Thiele, H., Nürnberg, P., Gärtner, J., Orsomando, G., and Coleman, M.P. (2019). Homozygous NMNAT2 mutation in sisters with polyneuropathy and erythromelalgia. *Exp Neurol* 320**,** 112958.

Lukacs, M., Gilley, J., Zhu, Y., Orsomando, G., Angeletti, C., Liu, J., Yang, X., Park, J., Hopkin, R.J., Coleman, M.P., Zhai, R.G., and Stottmann, R.W. (2019). Severe biallelic loss-of-function mutations in nicotinamide mononucleotide adenylyltransferase 2 (NMNAT2) in two fetuses with fetal akinesia deformation sequence. *Exp Neurol* 320**,** 112961.

Miyake, N., Fukai, R., Ohba, C., Chihara, T., Miura, M., Shimizu, H., Kakita, A., Imagawa, E., Shiina, M., Ogata, K., Okuno-Yuguchi, J., Fueki, N., Ogiso, Y., Suzumura, H., Watabe, Y., Imataka, G., Leong, Huey y., Fattal-Valevski, A., Kramer, U., Miyatake, S., Kato, M., Okamoto, N., Sato, Y., Mitsuhashi, S., Nishino, I., Kaneko, N., Nishiyama, A., Tamura, T., Mizuguchi, T., Nakashima, M., Tanaka, F., Saitsu, H., and Matsumoto, N. (2016). Biallelic *TBCD* mutations cause early-onset neurodegenerative encephalopathy. *Am J Hum Genet* 99**,** 950-961.

Nicita, F., Ginevrino, M., Travaglini, L., D'arrigo, S., Zorzi, G., Borgatti, R., Terrone, G., Catteruccia, M., Vasco, G., Brankovic, V., Siliquini, S., Romano, S., Veredice, C., Pedemonte, M., Armando, M., Lettori, D., Stregapede, F., Bosco, L., Sferra, A., Tessarollo, V., Romaniello, R., Ristori, G., Bertini, E., Valente, E.M., and Zanni, G. (2020). Heterozygous KIF1A variants underlie a wide spectrum of neurodevelopmental and neurodegenerative disorders. *J Med Genet* 58**,** 475-483.

Tataranni, T., and Piccoli, C. (2019). Dichloroacetate (DCA) and cancer: An overview towards clinical applications. *Oxid Med Cell Longev* 2019**,** 8201079.

Wrobel, L., Hill, S.M., Djajadikerta, A., Fernandez-Estevez, M., Karabiyik, C., Ashkenazi, A., Barratt, V.J., Stamatakou, E., Gunnarsson, A., Rasmusson, T., Miele, E.W., Beaton, N., Bruderer, R., Feng, Y., Reiter, L., Castaldi, M.P., Jarvis, R., Tan, K., Bürli, R.W., and Rubinsztein, D.C. (2022). Compounds activating

VCP D1 ATPase enhance both autophagic and proteasomal neurotoxic protein clearance. *Nat Commun* 13, 4146.
